# Obligatory and facilitative allelic variation in the DNA methylome within common disease-associated loci

**DOI:** 10.1038/s41467-017-01586-1

**Published:** 2018-01-02

**Authors:** Christopher G. Bell, Fei Gao, Wei Yuan, Leonie Roos, Richard J. Acton, Yudong Xia, Jordana Bell, Kirsten Ward, Massimo Mangino, Pirro G. Hysi, Jun Wang, Timothy D. Spector

**Affiliations:** 1 Department of Twin Research & Genetic Epidemiology, King’s College London, London, SE1 7EH UK; 2 MRC Lifecourse Epidemiology Unit, University of Southampton, Southampton, SO16 6YD UK; 3 Epigenomic Medicine, Biological Sciences, Faculty of Environmental and Natural Sciences, University of Southampton, Southampton, SO17 1BJ UK; 4 Human Development and Health Academic Unit, Institute of Developmental Sciences, University of Southampton, Southampton, SO16 6YD UK; 5 BGI-Shenzhen, Shenzhen, 518083 China; 6 Institute of Cancer Research, Sutton, SM2 5NG UK; 7 MRC London Institute of Medical Sciences, Imperial College London, Du Cane Road, London, W12 0NN UK

## Abstract

Integrating epigenetic data with genome-wide association study (GWAS) results can reveal disease mechanisms. The genome sequence itself also shapes the epigenome, with CpG density and transcription factor binding sites (TFBSs) strongly encoding the DNA methylome. Therefore, genetic polymorphism impacts on the observed epigenome. Furthermore, large genetic variants alter epigenetic signal dosage. Here, we identify DNA methylation variability between GWAS-SNP risk and non-risk haplotypes. In three subsets comprising 3128 MeDIP-seq peripheral-blood DNA methylomes, we find 7173 consistent and functionally enriched Differentially Methylated Regions. 36.8% can be attributed to common non-SNP genetic variants. CpG-SNPs, as well as facilitative TFBS-motifs, are also enriched. Highlighting their functional potential, CpG-SNPs strongly associate with allele-specific DNase-I hypersensitivity sites. Our results demonstrate strong DNA methylation allelic differences driven by obligatory or facilitative genetic effects, with potential direct or regional disease-related repercussions. These allelic variations require disentangling from pure tissue-specific modifications, may influence array studies, and imply underestimated population variability in current reference epigenomes.

## Abstract (web summary)

Genomic polymorphisms affect the epigenome, which in turn influences how epigenome- and genome-wide analysis are interpreted. Here, the authors characterise allelic differences in DNA methylation driven by obligatory or facilitative genetic effects, which may affect disease-related loci.

## Introduction

The hunt for disease-implicated genetic sequences is a major focus of medical research because it may reveal precise molecular insights into disease pathophysiology. Genome-wide association studies (GWAS) have successfully identified thousands of common genetic loci associated with human diseases and phenotypes^1^. However, linkage disequilibrium (LD) between variants, incomplete coverage and gaps in their functional annotations make it difficult to establish a firm causal and functional mechanism between the statistically identified variants and the phenotypes they associate with. Integration with disease-relevant and tissue-specific functional indicators or epigenetic marks within these regions, such as DNase I hypersensitivity sites (DHSs)^2^, histone modifications^3, 4^ and DNA methylation variation^5, 6^, can highlight candidate active variants. This dissection of GWAS signals enables progress from associated SNP to mechanistic understanding^7, 8^.

Epigenetic variation in relation to genome sequence falls into three main categories: ‘pure’ if under no genetic influence, ‘facilitated’ when genetic polymorphism enable variability and ‘obligatory’, if sequence variants directly predict the epigenetic state^9^. Thus, defining how epigenomes vary with respect to genetic influence and the mechanistic role this plays over phenotypic expression can greatly increase understanding of genetic regulation in heath and in disease. Genetic influences on the epigenome, such as enhancer variation, are strong enough to be observed in only 19 diverse ancestry individuals^10^.

Here we report an analysis of the relationship between allelic dosage of genetic risk SNPs that have previously been significantly and reliably associated with human phenotypic variation (via the NHGRI-EBI GWAS Catalogue^1^) and DNA methylation within the LD block harbouring it. These GWAS regions are not only robustly associated with human traits but are also known to be enriched for functionality^7, 11^. We used genome-wide methylation-dependent immunoprecipitation second-generation sequencing (MeDIP-seq) data derived from peripheral blood and high-quality SNP array genotype data from 3128 samples of European ancestry. Therefore, we broadly capture DNA methylation differences between risk and non-risk GWAS haplotypes, or haplotype-specific DNA methylation (HSM) variation^12^. The underlying strong extent of genetic influences on the epigenome is increasingly acknowledged^13^. CpG density is fundamental in defining the background DNA methylome, along with cell-specific usage of available binding sequences for transcription factors^14^. Common population variation in CpGs is significant via SNPs within this dinucleotide (CpG-SNPs) as well as larger variants. Recent base-resolution data has further supported that regional epigenetic effects exist within the DNA methylome^15^. We took advantage of the fact that this immunoprecipitation-derived signal is strongly influenced by the number of methylated cytosines in the DNA fragment^16^ to capture these regional allelic variation effects, and can be considered more akin to ChIP-seq than base-resolution analyses. This leads to a distinct signal of population variation in both facilitated and obligatory genetic effects on the DNA methylome. Allelic variations in genetic dosage effects on this DNA methylation score are allelic signal differences not epigenetic variability. However, these may as well contribute to regional or neighbouring allelic epigenetic variation, with effects on traits, or even bias other analytical measures, including bisulphite arrays and sequencing.

In this study we identify robust alleic variation in DNA methylation signal within disease-related loci. This is contributed to, but not fully accounted for, by non-SNP variants. We show that these demarcated regions are, in fact, functionally enriched. Furthermore, CpG-SNP variation is important in this, through not only its fascilitative influence on signal, but also its impact on allele-specific DHS. This survey increases our understanding of common epigenomic variation, the extent of inter-individual genetic variation’s influence on the epigenome, and its potential relevance with regard to genetic common disease-related variation.

## Results

### DNA methylation variation by GWAS SNP risk carrier status

We investigated 8093 GWAS phenotype-associated results (*p < *1 × 10^−7^) listed in the NHGRI-EBI GWAS catalogue^1^. Due to some SNPs being associated with more than one trait, this amounted to 5474 unique SNP by GWAS LD block analyses. In addition, because of GWAS results co-locating in the same LD block, this reduced to a total of 2709 blocks, which cover 22.1% of the entire length of the genome.

**Fig. 1:**
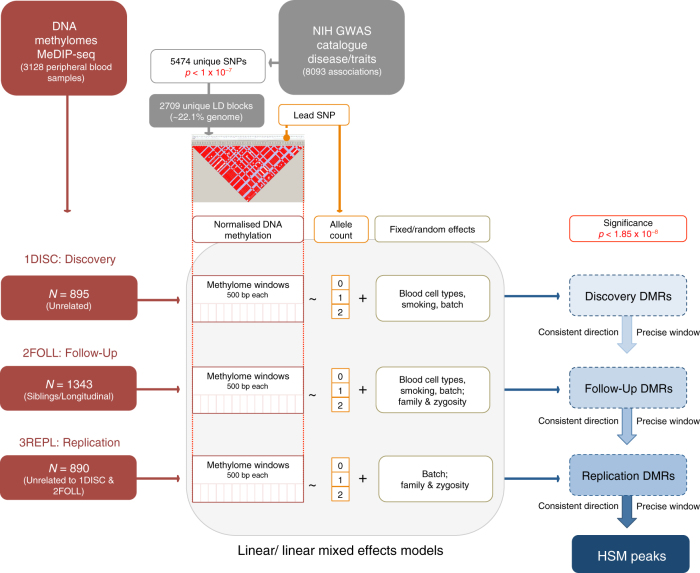
Study design. Flowchart illustrating the three separate analyses performed then collated to find consistent significant (*p* < 1.85 × 10^−8^, linear model, linear mixed model, see ‘Methods’) and directional changes in risk haplotype-specific DNA methylation (HSM) peaks. The three data sets comprise: 1DISC (*n* = 895), 2FOLL (*n* = 1343) and 3REPL (*n* = 890)

The total peripheral blood DNA methylome samples were split into three separate data sets (Fig. 1 and ‘Methods’). The first was the discovery analysis set (1DISC) which included 895 unrelated individuals. The second, follow-up data set (2FOLL, *N* = 1343) included 1343 siblings and additional time-point (longitudinal) samples from the individuals of 1DISC. The replication data set (3REPL) comprised 890 additional individuals, unrelated to any of the previous two sets. To identify differentially methylated regions (DMRs) between risk and non-risk GWAS haplotypes, the haplotype-specific DNA methylation (HSM) peak analysis assessed the linear relationship between the allelic count of the GWAS SNP and DNA methylation scores in 500 bp overlapping windows across the LD block. This included adjustments for critical covariates (Methods). Only HSM peaks retaining significance beyond multiple testing correction (*p* < 1.85 × 10^−8^, linear model or mixed effect model) in all three data sets and with a consistent direction of effect are reported here.

Our analysis is similar in concept but methodologically different to previous DNA methylation quantitative trait loci (mQTL) analyses that identify via array data significant SNP associations with DNA methylation^13, 17, 18^. We focus only on those genetic changes already robustly associated with disease via GWAS and, additionally, the use of MeDIP-seq data enables the entire related LD block regions to be interrogated, not isolated and predetermined CpGs. To identify obligatory or facilitated genetic effects on DNA methylation that are in strong LD with the GWAS SNP, we apply our analysis within the recombination-defined LD blocks, were these signals strongly reside (Supplementary Fig. 1). Whilst 'pure' epigenetic changes may exist beyond these limits, our focused approach dramatically increases our study power to find these genetically driven variations.

We identified a consensus set of 16,060 500 bp windows, which were then merged due to overlapping and adjacent locations into the final set of 7173 distinct HSM peaks (Supplementary Data 1). The overlap between significant peaks identified in 1DISC with 2FOLL was 88.1% and of these 82.0% overlapped with 3REPL. The HSM peaks in total span over ~5.86 Mb with an average genomic length of ~0.82 kb each (Supplementary Fig. 2).

### HSM peaks strongly overlap with genetic variants

**Fig. 2:**
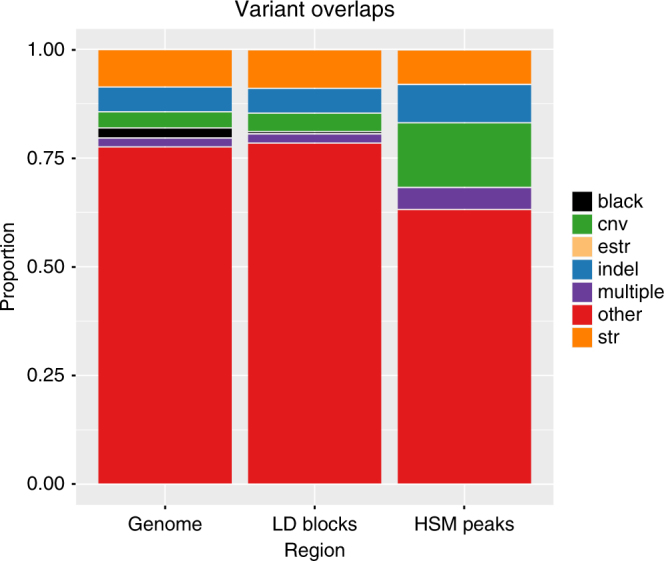
HSM peak overlap with genetic variants. Overlap of HSM peak significant windows with genetic variants in the Genome, GWAS LD block regions and the HSM peaks. Black=blacklist regions from ENCODE (removed from subsequent analysis); CNV (Green)=copy number variants from the database of genomic variants from Zarrei et al.^68^; STR (Orange)=short tandem repeats from Willems et al.^69^; eSTR (Fawn)=expression-associated short tandem repeats from Gymrek et al.^70^; Indel (Blue) from TwinsUK data set; multiple variant overlaps (Purple) and other category (Red) will comprise common SNPs which include CpG-SNPs

We subsequently investigated the overlap of these significant windows with known common genetic variants: copy number variants (CNVs), insertions and deletions (Indels) and short tandem repeats (STRs). 36.8% of the HSM peaks overlap these non-SNP variants or combinations of them. As would be expected there is strong proportional enrichment for non-epigenetic dosage effects driven by these CNVs (green), indels (blue), STRs (orange) and regions overlapping multiple variant types (purple) compared to their fractions both across the genome and within the GWAS LD block regions (Fig. 2, Fisher’s exact *p* < 2.2 × 10^−16^). The total count for overlap with the major variant classes with their *p*-value distributions is shown in Supplementary Fig. 3. Thus, whilst large-scale variants, such as CNVs, do have an increased impact within these regions, they do not account for the entire signal as 63.2% are apportioned to the ‘Other’ category.

**Fig. 3:**
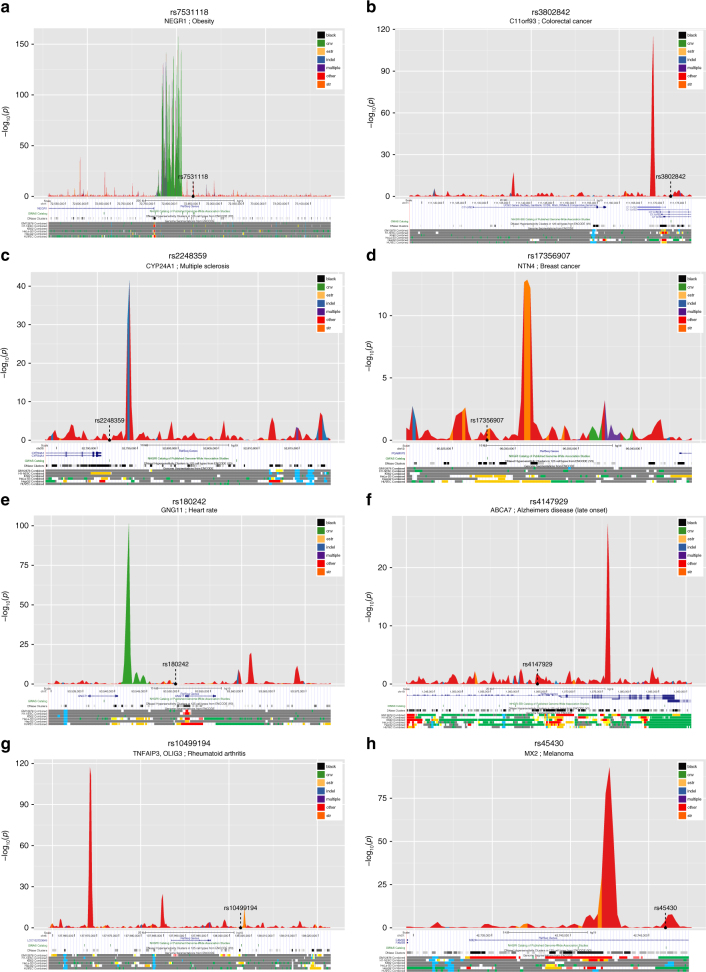
HSM peaks within selected GWAS LD block regions. The *y*-axis denotes the -log_10_(*p* value) of the differential DNA methylation according to the genotypic status for the index (GWAS associated) SNP and the *x*-axis is the genomic position (in base-pairs) along the LD block. The HSM peaks are coloured in accordance with the class of polymorphisms they overlap with, i.e. copy number variant (CNV, Green), short tandem repeats (STR, Orange), expression-associated short tandem repeats (e-STR, Fawn), insertion–deletions (Indels, Blue), multiple variant overlaps (Purple) and other category (Red). **a** *NEGR1* obesity locus; **b** intergenic colorectal cancer; **c** *CYP24A1* multiple sclerosis; **d** *NTN4* breast cancer; **e** *GNG11* heart rate; **f** *ABCA7* Alzheimer’s disease; **g** *TNFAIP3* rheumatoid arthritis and **h** *MX2* melanoma. Underneath is the UCSC browser track for location with RefSeq genes, GWAS catalogue SNPs, DNase-I hypersensitivity clusters and combined chromatin segmentation tracks. The segmentation tracks are in standard colours (Red: Promoter; Light Red: Promoter Flanking; Orange: Enhancer; Yellow: Weak Enhancer; Blue: CTCF element; Dark Green: Transcribed Region; Grey: Repressed/Low Activity)

To further illustrate these risk haplotype-specific DNA methylation effects we display eight examples of the 5474 GWAS SNP by DNA methylation HSM results in Fig. 3. The total set of graphs and results for all analysed GWAS SNPs are available for browsing and download at http://www.epigenome.soton.ac.uk/hsm/hsm.php.

**Fig. 4:**
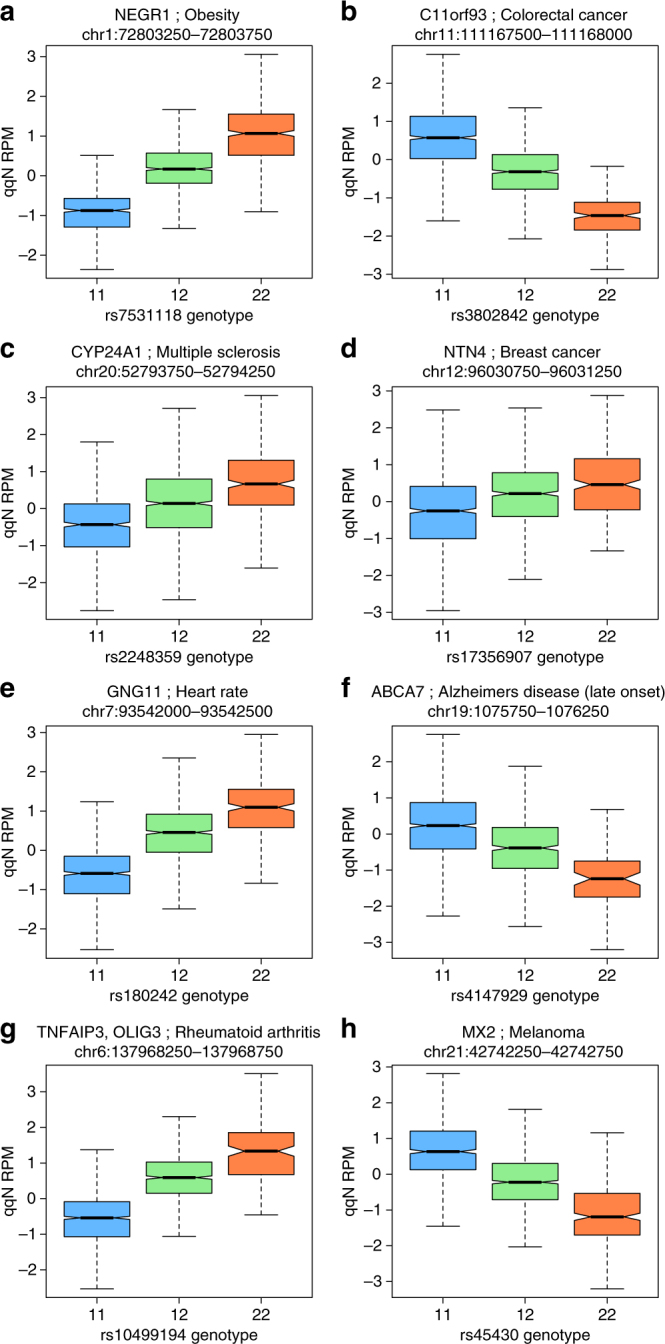
Variation by genotype within selected HSM peaks. Boxplots of most significant 500 bp windows within HSM peaks from within selected the GWAS LD block regions from Fig. 3. Normalised reads per million DNA methylation (*y*-axis: qqN RPM) within window by genotype for the GWAS SNP (*x*-axis: Genotype categories represented as 11, 12 and 22). **a** *NEGR1* obesity locus, rs7531118; **b** intergenic colorectal cancer, rs3802842; **c** *CYP24A1* multiple sclerosis, rs2248359; **d** *NTN4* breast cancer, rs17356907; **e** *GNG11* Heart Rate, rs180242; **f** *ABCA7* Alzheimer’s disease, rs4147929; **g** *TNFAIP3* rheumatoid arthritis, rs10499194 and **h** *MX2* Melanoma, rs45430

An extremely strong methylated signal difference due to dosage effects of a CNV is clearly seen in the known SNP-tagged 40 kb and 8 kb large CNV deletions near the obesity-locus *NEGR1* ^19^ (green, Fig. 3a). The *CYP24A1* multiple sclerosis locus^20^ shows an HSM peak overlapping a common indel (blue, Fig. 3c), the *NTN4* breast cancer locus^21^ with an STR (orange, Fig. 3d) and the *GNG11* locus associated with heart rate^22^ with a CNV (green, Fig. 3e). A partial STR overlap can also be seen with the *MX2* melanoma locus^23^ (orange, Fig. 3h). However, the SNP rs3802842 associated with colorectal cancer (Fig. 3b)^24^, as well as the *ABCA7* Alzheimer’s disease^25^ (Fig. 3f) and the *TNFAIP3* rheumatoid arthritis^26^ (Fig. 3g) loci, all possess strong signals that are not attributed to known CNV, Indel or STR co-localisations. The peak window differences in DNA methylation according to genotype of the GWAS SNP for these regions are shown in Fig. 4.

Furthermore, using a combined analysis for all 3128 samples together for DNA methylation versus only GWAS allelic count, without other covariates, clearly mimics the initial discovery set result, but with more power (Supplementary Fig. 4). This starkly displays how strong this over-riding genetic effect is on this analysis technique. The complete combined result had consistent overlap with the HSM peak set (7163 of the 7173; 99.9%). These strongly genetically associated DNA methylation changes may suggest potential pathophysiological mechanisms differentiating risk and non-risk GWAS haplotypes, if not already known, within these respective trait-related loci.

### CpG and CpG-SNP density is increased in HSM peaks

**Fig. 5:**
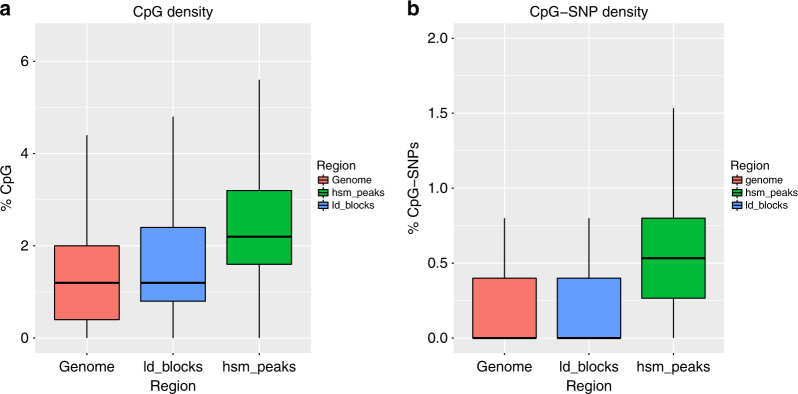
CpG and CpG-SNP density within HSM peaks. **a** CpG density and **b** CpG-SNP density within HSM peak regions compared to within GWAS LD block regions and genome. (Expressed as % of sequence, i.e. 50 CpGs in 100 bp = 100%.)

HSM peaks possess a higher CpG density (12.8 CpG/kb, or 2.55% of sequence is CpG dinucleotides) than both the background genome (1.84%) and the GWAS LD block regions (2.15%) (Fig. 5a, HSM peaks versus GWAS LD block regions, OR = 1.19, *p* < 2.2 × 10^−16^). However, they are predominately not within CpG-dense ‘CpG island’ regions (see later). There is also an increased number of SNPs within HSM peaks (6.60 SNPs/kb) compared to the GWAS SNP LD block regions (3.91 SNPs/kb, Fisher’s exact *p* < 2.2 × 10^−16^). Due to hypermutability of methylated cytosines, CpG-SNPs are a significant proportion of all SNPs and we calculated that 32.7% of common SNPs (MAF ≥ 1%) within the GWAS LD Block Regions are CpG-SNPs. This gives a background density of 0.26% CpG-SNP sequence in these regions, however, within HSM peaks they are found at more than twice this density (0.57%, OR = 2.25, Fisher’s exact *p* < 2.2 × 10^−16^, Fig. 5b).

A subset of 4482 HSM peaks, do not overlap with known CNV, Indel or STR (included in the miscellaneous ‘Other’ category for the purposes of our work). These are therefore expected to contain numerous SNPs, including methylation signal influencing CpG-SNPs^27, 28^. In fact, it is more than double, with 83.5% of SNPs being CpG-SNPs within these HSM peaks compared to the background of 40.1% in the ‘Other variant’ category windows within the GWAS LD block (OR = 7.66, Fisher’s exact *p* < 2.2 × 10^−16^). 92.6% of ‘Other’ HSM peaks contain a CpG-SNP with an average of 2.26 CpG-SNPs within these CpG-SNP containing peaks, thus indicating, as expected, that clusters of CpG-SNPs are strong drivers of HSM signal outside other known variants (Supplementary Fig. 5).

### HSM peaks are enriched for chromatin segmentation enhancers

**Fig. 6:**
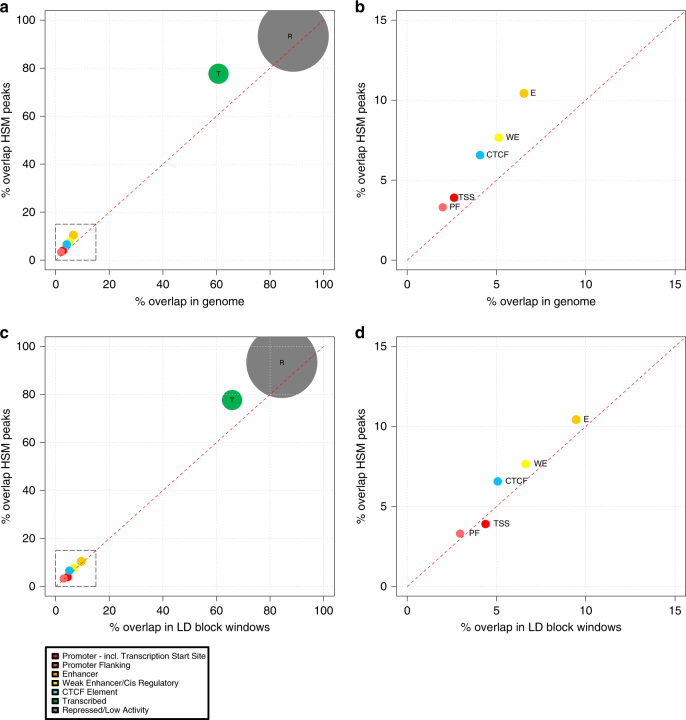
Combined chromatin segmentation enrichment. Overlap of HSM peaks with combined chromatin segmention functional annotations. Seven functional categories are included in the combined algorithm: PF promoter flanking, TSS transcription start site, CTCF, WE weak enhancer, E enhancer, T transcribed region and R repressed. Size of circle as a proportional adjustment to genome size (~10^(Region Proportion)^). **a** Proportional overlap with HSM peaks and Genome (non-overlapping 500 bp windows, minus blacklist regions). Highlighted region in grey dashed box displayed in **b**. **c** Proportional overlap with HSM peaks and GWAS LD blocks (non-overlapping 500 bp windows, minus blacklist regions). Highlighted region in grey dashed box displayed in **d**. Graphs are adapted from Epiexplorer^71^

We examined the overlap of the HSM peaks with Chromatin segmentation data from ENCODE in six tissues^4^ (Fig. 6). Due to the known functional enrichment of GWAS regions when compared to genome, these HSM peaks show very strong enrichment for all for these defined loci (Fisher’s exact all *p* < 1 × 10^−10^, Fig. 6a, b, Supplementary Data 2).

We then compared these regions specifically against their proportions within the GWAS LD blocks only. This was in order to test that the HSM peaks are not depleted exceptions within the functionally enriched GWAS regions. However, even comparing within the GWAS LD block regions (Fig. 6c, d), they show a small but significant enrichment for all categories, except for transcription start sites (TSS) and Promoter loci. This includes enhancer (Fisher’s exact *p* = 5.58 × 10^−3^), weak enhancer (Fisher’s exact *p* = 6.01 × 10^−4^) and CTCF loci (Fisher’s exact *p* = 2.58 × 10^−8^) as well as the large genomic regions of transcribed and repressed regions (Fisher’s exact both *p* < 2.2 × 10^−16^). Generalised MeDIP-seq coverage differences were compared via the proportion of zero coverage across the differing functional units and indicated no significant influence on our enrichment calculations (Supplementary Table 1).

Overall, 748 HSM peaks (~10.4%) overlap enhancer signal from this combined segmentation in at least one of these six tissues, which rises to 1089 HSM peaks (~15.2%) if weak enhancers are also included.

### HSM peaks are not functionally depleted

**Fig. 7:**
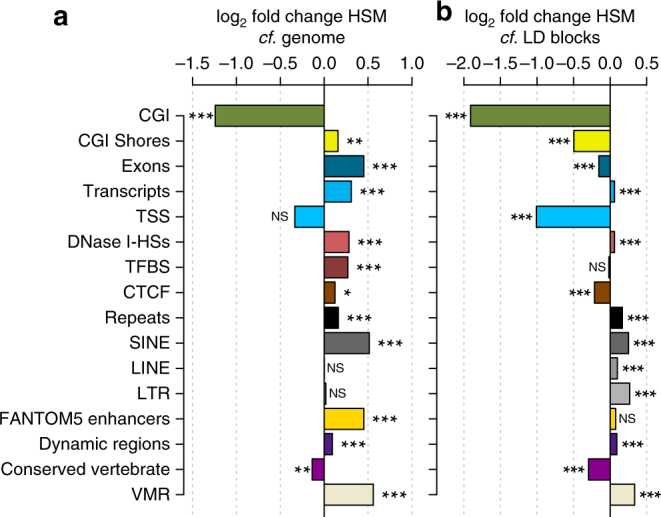
Functional enrichment for HSM peaks. Functional enrichments log_2_ fold change for HSM peaks in comparison to **a** genome and **b** GWAS LD block regions. These were compared with CpG Islands (CGI); CGI shores; exons; transcripts; transcription start sites (TSS); DNase-I hypersensitivity sites (DHS); transcription factor binding sites (TFBS); CTCF; repeats, including SINE, LINE and LTR subclasses; FANTOM5 enhancers; Dynamic DNA methylation regions; conserved vertebrate regions; variably methylated regions (VMRs). See ‘Methods’ for analysis and datasources. Fisher’s exact test, NS non-significant, *p < 0.05, **p < 0.01, ***p < 0.001

The HSM peaks were then overlapped with known genetic functional sets and compared again against both the entire genome and to the GWAS LD block regions (Fig. 7a, b, Supplementary Data 3). Firstly, 11 of these 16 functional categories show significant differential enrichment within the HSM peaks (Fisher’s exact at least *p* < 0.05) compared to the entire genome. By contrast CpG Islands (CGI) and vertebrate conserved regions show a significant depletion. Whilst we expect depletion within CGI of MeDIP-seq signal across the genome, as these regions are predominately unmethylated, the 32 CGI in which we do find GWAS-related DNA methylation variation are of obvious interest for those traits. This indicates potential genetic variability within these CGI, in LD with the GWAS SNP, which is strongly influencing their DNA methylation state and may have canonical CGI functional consequences. Overall, the HSM regions are, therefore, representative of the functionally enriched GWAS LD block regions they are derived from, including enrichment for Fantom5-derived enhancers, DHSs and CTCF elements (Fig. 7a).

Then compared to the average GWAS LD block regions themselves, the HSM regions show significant depletion in 6 of the 16 categories, (Fig. 7b), such as CGI shores, exons and CTCF loci. However transcripts, DHSs, all repeat classes, variably methylated regions (VMRs^29^), and dynamic regions^30^ all remain significantly enriched (all Fisher’s exact *p* < 1 × 10^−4^). Thus, there is strong enrichment across both of the base-resolution data sets used to identify regions of high DNA methylation variability, the VMRs, (54 DNA methylomes across 21 cell/tissue types^29^) and ‘Dynamic’ regions (24 developmental and primary cells^30^). This implies that genetic variation between samples may in fact be a significant contributor to the identified regions of increased DNA methylation variability within both these studies.

### CpG-SNPs enriched in allelic DHSs

DNase I hypersensitivity sites identify accessible regions of the genome and therefore act as broad functional indicators^31, 32^. As above, we found increased DHSs within HSM peaks. To further investigate the enrichment of CpG-SNPs we also identified in the HSM peaks, we explored SNPs that influence allele-specific DHSs. SNPs altering transcription factor (TF) binding were identified in a study by Moyerbrailean et al. by interrogating DHS footprinting data within the binding sites of 1372 TFs across 153 tissues^33^. About 66% of the ~5.8 million SNPs that reside within TF motifs were predicted to significantly modify binding. However, only 3217 SNPs demonstrated allele-specific differences in DHSs. Interestingly, we calculate that these allele-specific DHS SNPs are very strongly enriched for CpG-SNPs: 54.14% (1742) compared to the genome average of ~31.1% (OR=2.62, Fisher’s exact *p* < 2.2 × 10^−16^). This further points to the potential functional importance of CpG-SNPs that we and others have suggested previously^12, 34, 35^. 15 HSM peaks (~0.21%) overlap with these 3217 SNPs identified in this study by Moyerbrailean et al., and despite this small number, are enriched compared to the genome (OR=2.25, Fisher’s exact *p* = 1.93 × 10^−3^). 11 of these 15 SNPs (73.3%) are in fact CpG-SNPs, clearly fitting with their potential to directly influence allelic methylation and impact functionally.

### HSM peaks are not specifically blood tisssue enriched

**Fig. 8:**
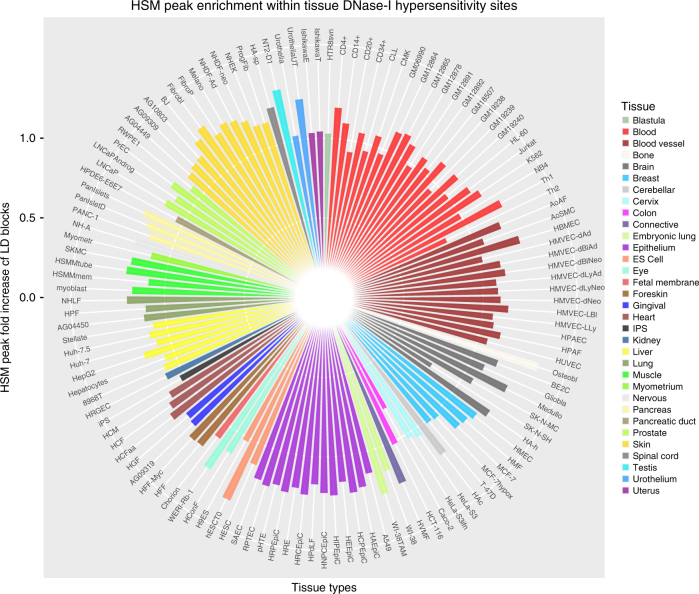
Tissue-specific DNase-I hypersensitivity site enrichment. Fold enrichment for overlap of HSM peaks with 125 tissue-specific DNase-I hypersensitivity site data (via ENCODE) compared to the genome. *Y*-axis indicates log_2_ fold enrichment

To interrogate the tissue-specific nature of our HSM peaks we compared their overlap with the DHS ENCODE data sets produced from 125 different tissue types. Our DNA methylome data set was derived from peripheral blood, but due to strong genetic influence, these HSM peaks are not specifically enriched for the blood-derived subset (22 of the 125) of DHSs (Fig. 8). In fact, a majority, 99 of the 125 tissue types, show at least nominal significant increase within the HSM peaks compared to genome (79.2%, *χ* ^2^ *p* < 0.05), across a range of different tissues (Supplementary Data 4). This, therefore, supports the genetic and non-tissue-specific systemic nature of the HSM peaks identified.

Additionally, we further explored for any evidence that our results are biased towards blood-related disorders using the 15 broad disease categories for GWAS associations classified by Maurano et al.^32^. We see no evidence for HSM peaks to reside within GWAS LD blocks of blood-specific or related diseases above any of the other categories, either by number of HSM peaks per GWAS LD block or size-corrected HSM peaks/kb (Supplementary Fig. 6).

### Facilitative transcription factor binding site enrichment

We identified 47 enriched motifs using MEME-ChIP^36^ within the HSM peak DNA sequences. These were compared against the JASPAR Vertebrates database for enriched transcription factor binding sites (TFBSs). Nineteen of these matched significantly with known sequences via the TOMTOM algorithm^37^ (Supplementary Data 5). Notably, this included the motifs for NRF1, ZFP161 and MYCN (Supplementary Fig. 7). These were recently identified by Domcke et al. to be methylation-sensitive TFs^38^, with NRF1 also shown to significantly influence nearby gene expression. These HSM peaks genetically introduce or include motifs for TFs were methylation can facilitate variability in binding and downstream action. Therefore, they support the potential for HSM peaks to play, not only an obligatory, but also in some cases a facilitative epigenetic role.

### HSM peaks are enriched for Alu, SVA and LTR repeat classes

This sequencing study as opposed to array analysis is able to obtain some preliminary indication of repetitive methylation variation. We further investigated the identified above enrichment for the SINE repeat class, which are predominately *Alu* repeats. This revealed that HSM peaks were enriched for the younger and more active *AluY* and *AluS* elements compared to the older *AluJ* element. This was compared to both the genome and GWAS LD block regions (OR 1.24 and 1.15, Fisher’s exact *p* = 5.81 × 10^−12^ and 7.82 × 10^−6^, respectively). These younger repeats still possess mobilisation ability^39^ and are more likely to be significant contributors to the population variation and haplotypic differences through direct and regional positional effects. Additionally, albeit smaller numbers, the even younger hominid-specific and *Alu*-containing SVA (SINE-VNTR-*Alu*) transposable element shows a significant enrichment (OR 2.08 and 1.44, Fisher’s exact *p* = 2.24 × 10^−6^ and 1.26 × 10^−2^, versus genome and GWAS LD block regions, respectively, Supplementary Fig. 8).

Within the LTR repeat class, HERV-H and LTR12C categories also show substantial overlap with our HSM peaks in comparison to the GWAS LD block regions background (OR 4.38 and 2.71, Fisher’s exact *p* < 2.2 × 10^−16^ and *p* = 7.82 × 10^−11^, respectively, Supplementary Fig. 8). Both these endogenous retroviruses (ERVs) are shown to be important in human development and the LTR12C subfamily has shown substantial co-location with enhancer signal that was tissue nonspecific^40^.

### Enrichment for allele-specific CTCF in HSM peaks

**Fig. 9:**
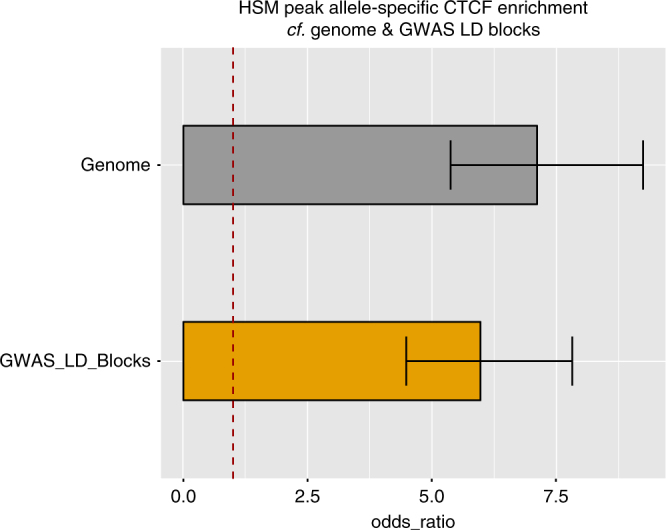
Allele-specific CTCF enrichment. Allele-specific CTCF (AS-CTCF) enrichment in HSM peaks compared to the genome and GWAS LD block regions. The AS-CTCF data is derived from Ding et al.^41^, which identified common human variants acting as quantitative trait loci (QTLs) that influenced binding in ChIP-seq CTCF data. Fisher’s exact test both *p* < 2.2 × 10^−16^, compared to genome and LD blocks, odds ratio (OR)=7.12 and 5.98, respectively. Error bars indicate 95% confidence intervals, dotted red line is OR=1

We further explored allele-specific functional differences using an allele-specific CTCF (AS-CTCF) data set from Ding et al.^41^ This study identified common human variants acting as quantitative trait loci (QTLs) that influenced binding in ChIP-seq CTCF data. They found 1837 AS-CTCF binding events across the genome and 26 of these overlap HSM peaks, an extreme enrichment compared by chance in comparison to the genome and even within GWAS LD block regions themselves (Fig. 9, OR = 7.12 and 5.98, both Fisher’s exact *p* < 2.2 × 10^−16^, respectively). This result suggests that these HSM peaks may be contributing to haplotypic variation in CTCF binding, potentially mediating population variation in 3D chromatin topography^42^. The haplotype allele-specific methylation (hap-ASM) results of Do et al. also identified a role for CTCF variability^6^. Furthermore, CTCF is known to commonly bind constitutively across many tissue types including a highly similar binding spectrum across all three germ layers in development^43^, which is consistent with the constitutive nature of the HSM peaks. Additional gene set enrichment analyses and multiple disease block identification are detailed in Supplementary Notes 1 and 2, with results in Supplementary Data 6 and 7, respectively.

### Allelic effects on DNA methylation array analysis

Excluding known common SNP effects on Illumina DNA methylation array probes is part of the standard technical quality control for epigenome-wide association study (EWAS) analysis^44^. This is generally performed with arbitrary thresholds for common SNPs located at the interrogated CpG and within 10 bp or up to 50 bp of the probe sequence, although more nuanced approaches are being explored^45^.

**Fig. 10:**
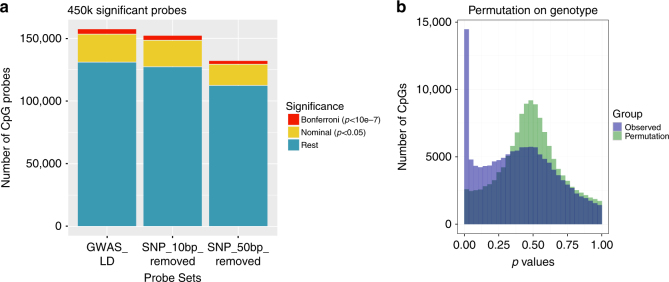
Significant Illumina 450k probes. **a** Number of 450k CpG probes within the GWAS LD block regions that show significant results for genetic effects as captured by haplotype-tagging lead SNP. Separated into all probes within the entire region (*n* = 157,473), and if probes pruned for those with SNPs within 10 bp (SNP_10bp_removed, *n* = 152,313) and 50 bp (SNP_50bp_removed, *n* = 132,221), respectively. Nominally significant results are shown in yellow and Bonferroni significant in red. **b** Observed *p*-values for CpG probes excluding those with common SNPs to 50 bp (in purple), versus results with the permutation of observed Genotype proportions (allelic count) (in green). This shows significant enrichment for low *p*-values in the observed data (empirical *p* < 0.01)

No allelic information is available from Illumina DNA methylation array results as methylation values are an average between both alleles. To investigate the potential genetic effects that may or may not be captured, we performed a similar analysis for possible allelic effects in GWAS LD blocks with a linear mixed model analysis in 811 Illumina 450k array peripheral blood-derived samples (88.9% overlap with MeDIP samples). The 450k array contain 169,151 probes (34.8%) within the GWAS LD block regions. After exclusion of multi-mapping probes, this reduced to 157,473 CpGs. Of these, 22,296 were nominally significant (*p* < 0.05) and 4192 were significant to a genome-wide Bonferroni level (*p* < 1 × 10^−7^, linear mixed model) for GWAS risk haplotype-capturing SNP allelic count. When common SNPs to 10 bp or 50 bp are excluded, these reduce to 21,091 and 16,878 at nominal, and 3890 and 2904 CpGs, at Bonferroni significance, respectively (Fig. 10a). Permutation analysis by random shuffling of observed genotype (allelic count) clearly displays the difference between observed and random results (Fig. 10b). 100× permutation strongly supported these findings as it identified only an average ~4524 (range 4290–4696) cytosines at nominal and 0.32 (range 0–2) at Bonferroni significance, respectively (empirical *p* < 0.01). These findings indicate the importance of mQTL and additional detailed genetic interrogation of array results.

## Discussion

We identified 7173 haplotype-specific methylation (HSM) peaks by investigating DNA methylation data in conjunction with robust GWAS SNPs and LD information. This analysis was performed in currently the largest available sequencing-based genome-wide DNA methylome data set. The identified loci are enriched for functional regions, such as enhancers, DHSs and allele-specific CTCF, illustrating their potential to play a pathogenic role.

This HSM analysis powerfully benefits from the combination of three main factors. Firstly, it focuses the analysis on functionally enriched^7, 11^ and robustly disease-associated GWAS LD block regions. Secondly, the MeDIP-seq data enables DNA methylation interrogation across the entire GWAS LD regions, not just predetermined CpGs. Array data does not provide a representative survey of population-level DNA methylome variation, its underlying genetic architecture^46^, and lacks the density of coverage required for powerful DMR calling^47^. When DMRs can be identified they are found to be strongly enriched for functional elements^30^. Thirdly, is the considerable reduction in the number of tests, by reducing these components to DNA methylation 500 bp windows and a single haplotype-tagging GWAS SNP within each LD block. Individual CpG by SNP analyses require a punitive multiple testing correction and our approach reduces this burden.

Our LD aware approach differs from many recent excellent studies exploring genetic influences on the DNA methylome due to its direct focus on known GWAS SNPs^6, 18, 35, 48^. Furthermore, whilst identifying ‘pure’ epigenetic changes by removing genetic effects has been the focus of some studies^6^, facilitative mechanisms are influential^35^. McClay et al. recently reported that the majority (75%) of *cis*-mQTL were due to collocating CpG-SNPs^35^, but only within GWAS regions were these functionally enriched. The individual genetic gain or loss of a CpG can impact upon dependent TF binding activity^38, 49^. It can also led to DNA conformation changes affecting protein–DNA interaction strength, including an order of magnitude increase in adjacent DHS^50^. We identified a strong enrichment of CpG-SNPs within HSM peak regions as well as within allele-specific DHS sites. Previous work from Jaffe et al.^51^ has also investigated GWAS loci with respect to DNA methylation from array data and identified a strong enrichment for mQTL within these loci. Furthermore, many of these mQTLs were not cell-type specific and involved critical disrupting CpG-SNPs, in similar fashion to our HSM data set. That analysis also proposed that many GWAS variants and highly correlated proxy SNPs influence nearby DNA methylation and in this way impact upon disease risk. An analysis in lung tissue by Shi et al. equally found that *cis*-mQTL reside at CpG-SNPs distal to both genes or CpG-dense promoter regions^52^, and that mQTLs themselves are also enriched for CTCF loci, DHS and chromatin modifications. In our analysis a strong enrichment particularly for allele-specific CTCF was also seen, potentially indicating the ability of these loci to contribute to population variation in 3D structure^42^.

On average ~2.65 HSM peaks were identified per GWAS LD blocks regions (59.0%≥1) and these will enable further hypotheses to be proposed in these disease susceptibility regions. The strong genetic influences on the DNA methylome leads to many more significant results than are seen for other potentially ‘pure’ epigenetic changes. For example, only 71 Bonferroni significant ageing-specific DMRs were identified in this same data set (including an LD correction for genetic effects)^53^. However, it needs to be clearly noted that methylation is the default state for the majority of CpGs throughout the genome. Those signals driven by variation in CpG number between risk and non-risk haplotypes can not be directly interpreted as functional, as is the case for tissue-specific epigenomic data, such as DHSs^32^. Yet the HSM peaks identified are enriched for functional indicators such as enhancer signal as well as motifs for DNA methylation sensitive TFs. Similarly to the hap-ASM results of Do et al.^6^ they are also located outside CpG island promoter regions. Some of these peaks may represent population variation in distal regulatory regions, such as low methylation regions (LMRs)^14^. These LMR loci require a genetic infrastructure of a slightly above baseline CpG level and co-locate with transcription factor binding and enhancer evidence. This may then facilitate time- or tissue-specific epigenetic variability.

Global diversity in large deletions, duplication and CNVs have had a significant influence on shaping the genome of human populations through selection, mutation and demography^54^. The functional potential of these large-scale variants is significant and these regions, as would be expected due to modulation of available CpGs, influence signal and strongly overlap with HSM peaks. Additionally, STRs contribute to HSM peaks and these variants have shown enhancer activity^55^ as well as the ability to influence expression^56^. Furthermore, DNA methylation within transposable elements can influence nearby gene expression^57^. Our data indicated enrichment for functionally implicated LTR repeat elements, HERV-H and LTR12C^40^. As well we see enrichment for younger primate-specific *Alu*s and hominid-specific SVA repeats, which have known germline insertion mobility and population variability^58^. Thus, some of the HSM peaks represent their direct or adjacent positional effects on the regional epigenome^58^.

GWAS results are capturing haplotypes and a single variant may not be the only causal element. Recent analysis has proposed that the fraction of causal variation tagged by common SNPs is higher than previously calculated^59^. Therefore, there may be multiple genetic variants impacting to varying degrees, with some acting via this observed DNA methylation variability. Whilst functional overlap with HSM peaks was identified, this may itself be an underestimate, as the data sets for comparison themselves, such as chromatin segmentation, have only been performed in small numbers^46^. Population epigenomic variation will exist beyond that in current reference epigenomes. Our evidence of genetic effects in both the VMR and ‘Dynamic’ methylome data further imply that genetic polymorphism drives some of these findings assumed to be purely epigenetic. Additionally, strong HSM effects were previously identified in the *FTO* GWAS locus^12^. This located a peak within an enhancer region, which influences *IRX3* expression^60^, that highlighted a SNP (rs7202116) subsequently found in a large meta-analysis to influence trait variability^61^. Thus, the potential of all 7173 HSM peaks is worthy of exploration and make excellent candidates for further functional analyses.

These extremely strong HSM signals clearly point to the caution and extra exploration required in the interpretation of population or non-inbred MeDIP as well as other DNA methylome analyses. Adjusting for known genetic influences impacts greatly on EWAS array analysis for common disease and phenotypes^47^. It is notable the pausity of significant directional disease-associated findings in robustly controlled experiments that have reduced genetic and cell-type heterogeneity issues by the use of isolated cell types in monozygotic twin discordant models^62^. Therefore, researchers need to be as aware as possible of the potential genetic confounding and/or interaction, both directly or due to regional or neighbouring effects. Our data set adds to the available genetic effects to be integrated in these analyses (Supplementary Data 1). Researchers should also interrogate results for additional evidence of strong genetic effects, such as ʻgap hunting’ in DNA methylation data that may indicate the strongest of these genetic influences^63^, as well as population-specific data availability of CpG-SNPs and larger genetic variants. A focused examination for mQTL SNPs influencing DNA methylation variability through haplotypic effects may be made more powerful by reducing tests through population-specific LD information. A tagging analysis may reveal unknown regional or haplotypic effects on the observed DNA methylation. We identified ~10.0% of the 450k CpG probes within these GWAS regions may be influenced (*p* < 0.05) by their lead SNP captured haplotype background, even after excluding probes with common SNP co-location. Identified effects require exploration for evidence of obligatory, facilitative, or dosage factors, as previously observed in array analysis^64^. Do et al. also identified haplotypic effects on a DNA methylation array analysis of Alzheimer’s disease being driven by the nearby genetic susceptibility factor at HLA-DR*^6^. Furthermore, of the 278,873 probes on the new Illumina EPIC (850k) within these GWAS regions, 1815 directly overlap an identified HSM peak, and this further rises to 6671 probes including those within a 1 kb flanking region. Technological advances, including direct assessment of DNA modifications in long reads to enable more robust genetic and epigenetic haplotypic assessment will obviously improve our knowledge of these interactions and their interplay in disease risk.

We identified functionally enriched DNA methylome variation between risk and non-risk GWAS haplotypes. This robust set of HSM peaks propose potential new mechanisms to combine with tissue-specific data to further understand these diseases. Thus, this integrative analysis is a step in the process of defining population variation in the facilitative and obligatory DNA methylome. Complete allelic integration of both genetic and epigenetic variability will be required to fully understand these human disease-associated regions. Future advances with direct epigenetic detection and longer reads, will help define more precisely this variability. These HSM peaks begin this process by giving strongly demarcated loci across these GWAS regions, for further exploration and integration in human disease susceptibility understanding.

## Methods

### Subjects

For this study, peripheral blood-derived DNA was provided by the adult volunteers from the UK Twin Register (TwinsUK Resource) who are rigorously phenotyped at visits at St Thomas’ Hospital, London. Blood is collected for haematological analysis and DNA extraction at these appointments. Tobacco smoking is queried at this time or via questionnaire within the nearest 5 years. Samples are stored at −80 °C in EDTA tubes before extraction via the Nucleon Genomic DNA Extraction Kit. DNA is subsequently stored in TE Buffer at −20 °C. The majority of samples have full blood count data. Twinning questionnaire determines twin zygosity and is confirmed by genotyping. Ethics were approved by Guy’s & St Thomas’ NHS Foundation Trust Ethics Committee (EC04/015—15-Mar-04) and written informed consent was obtained from all subjects in accordance with this.

The initial Discovery analysis set (1DISC) comprised of 895 DNA methylomes of 895 unique individuals. These included only a single sample from each family i.e. non-related individuals and only female samples to exclude sex-specific variation in DNA methylation. The second Follow-Up data set (2FOLL) comprised of 1343 methylomes and included siblings and some longitudinal data set from Set 1. The third Replication data set (3REPL) comprised 890 DNA methylomes of non-related individuals to the first two sets. Our discovery set is well powered to detect moderate sized effects (Cohen’s *f* ^2^ = 0.15)^65^ as we estimate these can be identified with 95% power in ~500 samples. Furthermore, we possess the added benefit in this analysis of the two additional data sets for conformation and replication.

### MeDIP-seq

The methods and analysis pipeline made use of Methylation Dependent Immunoprecipitation Illumina second-generation sequencing (MeDIP-seq). BGI-Shenzhen (Shenzhen, China) performed DNA sample preparation, followed by Sonication with a Covaris system (Woburn, MA, USA) that fragmented whole peripheral blood DNA. Then the MeDIP reaction was performed and 5 ug of fragmented genomic DNA was used for sequencing library preparation. Illumina Single-End DNA Sample Prep kit was employed and end repair, < A > base addition and adaptor ligation steps were performed. Adaptor-ligated DNA was incubated with an antibody for 5-methylcytosine (5mC) Cat. No.: CO2010021 mc-magme-048 from Diagenode (Liège, Belgium). The protocol for the MagMeDIP kit (mc-magme-048) was followed: combining 0.5 μl antibody + 0.5 μl water; then add 0.6 μl MagBuffer A, 1.4 μl water, 2 μl MagBuffer C, therefore resulting in a final volume of 5 μl for the IP reaction. Immunocapture was performed with magnetic bead conjugation to capture the enriched DNA fraction. Quantitative PCR validated this resultant MeDIP, which was then purified with Zymo DNA Clean & Concentrator-5 (Zymo Research), and amplified with adaptor-mediated PCR. Gel excision for size selection of fragments (200–500 bp) was performed and these were quality assessed by Agilent BioAnalyzer (Agilent Technologies, Santa Clara, CA, USA). The libraries were subjected to highly parallel 50-bp single-end sequencing on the Illumina HiSeq2000 platform. FASTQC (v0.10.0) assessed initial base composition QC successfully. MeDIP-seq data was aligned with BWA (>mapping quality score of Q10), and duplicates were removed. The average high-quality BWA aligned reads was ~16.9 million per sample. Quality control with SAMTools and FastQC and for MeDIP-specific analysis MEDIPS(v1.0)^66^ was used to produce reads per million (RPM). These data were produced as BED files of genomic windows (500 bp, 250 bp slide). Additional quality checks were also employed with Principle Components Analysis and correlation matrix, hierarchical clustering, dendogram, heatmap, and density plots. All analysis and co-ordinates cited are for genome build *Homo sapiens* hg19/GRCh37.

### GWAS linkage disequilibrium blocks

Linkage disequilibrium blocks of the SNPs in the NHGRI-EBI GWAS catalogue^1, 11^ were ascertained from the GRCh37 genetic map, downloaded from Center of Statistic Genetics, University of Michigan, Locuszoom 1.3^67^. Recombination rate 10 cM/Mb block boundaries were used. The NHGRI-EBI GWAS catalogue as at December 2014 provided the 8093 GWAS SNPs with *p* value<1 × 10^−7^ deposited to that time point. These are in fact 5522 unique individual SNPs due to co-associations for the same SNP. Within the above-identified LD blocks 5474 of these SNPs were positioned and due to SNPs co-locating in the same block these represented a total of 2709 blocks, covering ~22.1% of the genome.

### Risk haplotype-specific methylation analysis

DNA methylation within the LD blocks of the GWAS catalogue association SNPs was compared by linear regression with respect to allelic count of the haplotype-tagging SNP, termed HSM analysis^12^. This identifies broad DNA methylation differences between risk and non-risk GWAS haplotypes. With MeDIP there is a direct relationship between the number of methylated cytosines in the DNA fragment and the amount of DNA captured by the antibody^16^. Therefore, genetic gains or losses of CpGs will influence the enrichment of fragments strongly. Consequently, this analysis results in a distinct signal due to population variation in both facilitated and obligatory genetic effects on the DNA methylome. These regions may have direct or regional impacts on further surrounding CpG methylation states with the potential for significant functional effects in these GWAS regions. DNA methylation was scored within 500 bp windows with 250 bp overlap by normalised RPM for each window. In the discovery set (1DISC) a linear model was fitted including chronological age at blood extraction for DNA sample, blood cell subtypes (lymphocytes, monocyte, neutrophil and eosinophil), smoking status and batch. In the Follow-Up (2FOLL), a linear mixed effect model was used for allelic count to DNA methylation with the fixed effects of 1DISC with additional family and zygosity as random effects. The replication (3REPL) set analysis was the same as for 2FOLL, but included sex and excluded blood and smoking covariate information. The described two linear effects models were compared with null models that excluded allelic count by the ANOVA function by likelihood ratio test for calculation of *p*-values. The lme4 R package was used to perform the linear mixed effect analysis of the relationship between allelic count of the haplotype-tagging SNP and normalised DNA methylation assayed by MeDIP-seq.

To correct for multiple testing, a strict Bonferroni cut-off was calculated by the total number of DNA methylome windows tested in the analysis, 2,708,462. Thus, a *p*-value significance level of <1.85 × 10^−8^. The mean *p*-value was calculated for each window for GWAS LD block regions containing greater than one GWAS SNP. ENCODE poor mappability blacklist regions^31^ were subsequently removed from any further interpretation (13,726 windows removed). To identify a robust set of HSM peaks we determined those windows that passed the Bonferroni threshold in all three (1DISC, 2FOLL and 3REPL) subsets. The R (3.0.0) environment was used for all analysis, with graphing via the ggplot2 package with results and code available at http://www.epigenome.soton.ac.uk/hsm/hsm.php.

### Variants within GWAS LD block regions and HSM peaks

Common genetic variants that overlapped locations within the GWAS LD block regions were defined. This included copy number variants (CNVs), insertions and deletions (Indels) and short tandem repeats (STRs). As above the known Blacklist regions were overlapped (13,726 windows, ~0.5%) and then removed before subsequent enrichment analyses below. Common CNV data was ascertained from the Stringent set of the copy number variation map of the human genome in the Database of genomic variants from Zarrei et al. which includes CNV sized 50 bp to 3 Mb^68^. Indel data was obtained from the TwinsUK data set with 1000 Genomes imputation of MAF>0.05. Short tandem repeats or Microsatellite data was obtained from the landscape of human STR variation from Willems et al. of 689,512 STRs^69^. Furthermore, a subset of these STRs with recent evidence of effects on gene expression, 2060 expression STRs (eSTRs), was also investigated for overlap^70^. Large study numbers reduced substantially the potential influence of rare or private CNVs, indels and STRs on this analysis.

### CpG-SNP identification

The dbSNP build 142 common SNP data set was downloaded including SNP alleles and surrounding base sequence. This represents 12,449,124 common SNPs found in ≥1% of samples within autosomes. These were then interrogated for those that were CpG-SNPs, i.e. where the allelic variation created or abrogated a CpG dinucleotide. 3,873,489 (~31.1%) of these SNPs were determined to be CpG-SNPs.

### Enrichment analysis

We used Epiexplorer for our first examination of the HSM peaks^71^ for assessment of enrichment for chromatin state (ChromHMM), histone modifications and TFBSs. We downloaded the additional Combined segmentation data for 6 tissue types (Gm12878; H1hesc; Helas3; Hepg2; Huvec; K562) from UCSC. Additional functional enrichments were also downloaded from UCSC data on CpG islands, ENCODE DHS in 125 cell types^2^, Vertebrate Multiz Alignment and Conservation (100 Species) from 100Vert_El_phastConsElement100way bedfile (~10.1 m regions), and TFBSs from ENCODE v3 (690 data sets from wgEncodeRegTfbsClusteredV3^31^). ‘Dynamic’ DNA methylation regions were taken from Ziller et al.^30^ and eRNA validated FANTOM5 enhancers regions from Anderson et al.^72^. BEDTools (v 2.17.0)^73^ IntersectBed with the f -0.1 parameter tested overlap between 500 bp non-overlapping windows of these elements and within the GWAS LD block regions.

Genomic regions enrichment of annotations tool (GREAT v3.0.0) was used for genomic-space aware Gene and Functional pathway enrichments^74^. The binomial analysis with default setting for basal, and extension parameters (constitutive 5.0 kb upstream, 1.0 kb downstream and up to 1 Mb max extension) was employed. Curated regulatory domains were also included. Two background sets were used for comparison, firstly the entire genome and secondly the GWAS LD block regions (Supplementary Note 1).

Transcription factor binding site motif enrichment was performed with the JASPAR core 2014 vertebrates database in the MEME suit (MEME-ChIP^36^) with TOMTOM^37^ (v4.10.2) using FASTA sequence files for the HSM peaks. MEME-Chip analysis compared with a set of 1434 DNA motifs, between 5 and 30 in length (average length 13.6), from the database Vertebrates (in vivo and in silico).

### Tissue-specific and multi-tissue disease investigation

DNase I hypersensitivity sites data in 125 cell types, including 22 blood cell data sets, from the ENCODE analysis of Thurman et al.^2^ were downloaded to compare any potential tissue enrichment of the HSM peaks. The total basepair overlap with DHS sites was compared between the HSM peaks and the entire genome. To investigate whether these peripheral blood-derived HSM peaks were enriched for blood-related disease categories, the broad disease categories as defined in Maurano et al. were used which had classified 5655 SNP-trait associations^32^. These 15 classes are: aging; autoimmune disease; cancer; cardiovascular; diabetes; drug metabolism; haematological parameters; kidney, lung and liver; miscellaneous; neurological and behavioural; parasitic and bacterial disease; quantitative traits; radiographic parameters; serum metabolites and viral disease. This data set was also used to identify multi-tissue associations (Supplementary Note 2).

### DNA methylation array analysis

A data set of 811 females (two batches: 388 and 423) were analysed by the Infinium Human Methylation450 BeadChip from bisulphite-converted DNA derived from peripheral blood. 88.9% of these samples also overlap the MeDIP samples. The quality control steps comprised the removal of probes that reside on the X or Y chromosomes (*n* = 11,650), where the 50 bp sequence aligned to multiple locations in the genome (*n* = 17,764), or failed detection in ≥1 sample and with a bead count <3 in >5% of the samples. This resulted in a data set of 450,077 probes. Further QC involved inspection for outliers using boxplots for mean and median DNA methylation across all CpG sites, *β* density plots, and heatmaps. The proportion of blood cell subtypes was deconvoluted for CD8+ T cells, CD4+ T cells, B cells, Natural Killer cells, granulocytes and monocytes^75^. To correct for probe type bias all data was normalised via BMIQ^76^. Probes that resided within the GWAS LD Block Regions (169,151) were assessed for any significant GWAS SNP associated differentially methylated positions. This was performed using a linear mixed effects model fitted on standardised *β* values per probe (*N*(0,1)), with genotype as allelic count, age, smoking status, beadchip, position on the beadchip, granulocytes, monocytes, and CD8+ T cells as fixed effects, as well as family and zygosity as random effects. To assess for significance, ANOVA was used to compare this model to a null model without allelic count. Permutation was performed in R by random shuffling of genotype assignment of individuals whilst retaining all other variables constant.

### Allele-specific data sets

We accessed the data on allele-specific CTCF from Ding et al.^41^. Allele-specific DNase-I Hypersensitivity SNPs, also termed ‘Switch-SNPs’, influence TF binding and were downloaded from Moyerbrailean et al.^33^.

### Data availability

The data supporting the results of this article are available in the EMBL-EBI European Genome-phenome Archive (EGA) under Data set Accession number EGAD00010000983 (https://www.ebi.ac.uk/ega/datasets/EGAD00010000983).

## Electronic supplementary material

Supplementary Information

Peer Review File

Description of Additional Supplementary Files

Supplementary Data 1

Supplementary Data 2

Supplementary Data 3

Supplementary Data 4

Supplementary Data 5

Supplementary Data 6

Supplementary Data 7

## References

[1] Welter D (2014). The NHGRI GWAS catalog, a curated resource of SNP-trait associations. Nucleic Acids Res..

[2] Thurman RE (2012). The accessible chromatin landscape of the human genome. Nature.

[3] Ernst J (2011). Mapping and analysis of chromatin state dynamics in nine human cell types. Nature.

[4] Hoffman MM (2013). Integrative annotation of chromatin elements from ENCODE data. Nucleic Acids Res..

[5] Gagliano Sarah A (2016). Allele-skewed DNA modification in the brain: relevance to a schizophrenia GWAS. Am. J. Hum. Genet..

[6] Do C (2016). Mechanisms and disease associations of haplotype-dependent allele-specific DNA methylation. Am. J. Hum. Genet..

[7] Schaub MA, Boyle AP, Kundaje A, Batzoglou S, Snyder M (2012). Linking disease associations with regulatory information in the human genome. Genome Res..

[8] Claussnitzer M (2015). FTO obesity variant circuitry and adipocyte browning in humans. N. Engl. J. Med..

[9] Richards EJ (2006). Inherited epigenetic variation--revisiting soft inheritance. Nat. Rev. Genet..

[10] Kasowski M (2013). Extensive variation in chromatin states across humans. Science.

[11] Hindorff LA (2009). Potential etiologic and functional implications of genome-wide association loci for human diseases and traits. Proc. Natl. Acad. Sci. USA.

[12] Bell CG (2010). Integrated genetic and epigenetic analysis identifies haplotype-specific methylation in the FTO type 2 diabetes and obesity susceptibility locus. PLoS ONE.

[13] Chen L (2016). Genetic drivers of epigenetic and transcriptional variation in human immune cells. Cell.

[14] Schubeler D (2015). Function and information content of DNA methylation. Nature.

[15] Libertini E (2016). Information recovery from low coverage whole-genome bisulfite sequencing. Nat. Commun..

[16] Okitsu CY, Hsieh CL (2015). Sensitivity and specificity of immunoprecipitation of DNA containing 5-Methylcytosine. BMC Res. Notes.

[17] Gibbs JR (2010). Abundant quantitative trait loci exist for DNA methylation and gene expression in human brain. PLoS Genet..

[18] Gaunt TR (2016). Systematic identification of genetic influences on methylation across the human life course. Genome Biol..

[19] Wheeler E (2013). Genome-wide SNP and CNV analysis identifies common and low-frequency variants associated with severe early-onset obesity. Nat. Genet..

[20] Sawcer S (2011). Genetic risk and a primary role for cell-mediated immune mechanisms in multiple sclerosis. Nature.

[21] Michailidou K (2013). Large-scale genotyping identifies 41 new loci associated with breast cancer risk. Nat. Genet..

[22] Nam RK (2011). *New variants at 10q26* and *1*5q21 are associated with aggressive prostate cancer in a genome-wide association study from a prostate biopsy screening cohort. Cancer Biol. Ther..

[23] Barrett JH (2011). Genome-wide association study identifies three new melanoma susceptibility loci. Nat. Genet..

[24] Tenesa A (2008). Genome-wide association scan identifies a colorectal cancer susceptibility locus on 11q23 and replicates risk loci at 8q24 and 18q21. Nat. Genet..

[25] Lambert JC (2013). Meta-analysis of 74,046 individuals identifies 11 new susceptibility loci for Alzheimer’s disease. Nat. Genet..

[26] Plenge RM (2007). Two independent alleles at 6q23 associated with risk of rheumatoid arthritis. Nat. Genet..

[27] Schalkwyk LC (2010). Allelic skewing of DNA methylation is widespread across the genome. Am. J. Hum. Genet..

[28] Shoemaker R, Deng J, Wang W, Zhang K (2010). Allele-specific methylation is prevalent and is contributed by CpG-SNPs in the human genome. Genome Res..

[29] Gu J (2016). Mapping of variable DNA methylation across multiple cell types defines a dynamic regulatory landscape of the HumanGenome. G3 (Bethesda).

[30] Ziller MJ (2013). Charting a dynamic DNA methylation landscape of the human genome. Nature.

[31] Bernstein BE (2012). An integrated encyclopedia of DNA elements in the human genome. Nature.

[32] Maurano MT (2012). Systematic localization of common disease-associated variation in regulatory DNA. Science.

[33] Moyerbrailean GA (2016). Which genetics variants in DNase-seq footprints are more likely to alter binding?. PLoS Genet..

[34] Dayeh TA (2013). Identification of CpG-SNPs associated with type 2 diabetes and differential DNA methylation in human pancreatic islets. Diabetologia.

[35] McClay JL (2015). High density methylation QTL analysis in human blood via next-generation sequencing of the methylated genomic DNA fraction. Genome Biol..

[36] Machanick P, Bailey TL (2011). MEME-ChIP: motif analysis of large DNA datasets. Bioinformatics.

[37] Gupta S, Stamatoyannopoulos JA, Bailey TL, Noble WS (2007). Quantifying similarity between motifs. Genome Biol..

[38] Domcke S (2015). Competition between DNA methylation and transcription factors determines binding of NRF1. Nature.

[39] Bennett EA (2008). Active Alu retrotransposons in the human genome. Genome Res..

[40] Leung D (2015). Integrative analysis of haplotype-resolved epigenomes across human tissues. Nature.

[41] Ding Z (2014). Quantitative genetics of CTCF binding reveal local sequence effects and different modes of X-chromosome association. PLoS Genet..

[42] Tang Z (2015). CTCF-mediated human 3D genome architecture reveals chromatin topology for transcription. Cell.

[43] Tsankov AM (2015). Transcription factor binding dynamics during human ES cell differentiation. Nature.

[44] Price EM (2013). Additional annotation enhances potential for biologically-relevant analysis of the Illumina Infinium HumanMethylation450 BeadChip array. Epigenetics Chromatin.

[45] Naeem H (2014). Reducing the risk of false discovery enabling identification of biologically significant genome-wide methylation status using the HumanMethylation450 array. BMC Genomics.

[46] Taudt A, Colome-Tatche M, Johannes F (2016). Genetic sources of population epigenomic variation. Nat. Rev. Genet..

[47] Liu Y (2013). Epigenome-wide association data implicate DNA methylation as an intermediary of genetic risk in rheumatoid arthritis. Nat. Biotechnol..

[48] van Dongen J (2016). Genetic and environmental influences interact with age and sex in shaping the human methylome. Nat. Commun..

[49] Yin, Y. et al. Impact of cytosine methylation on DNA binding specificities of human transcription factors. *Science* **356**, eaaj2239 (2017). 10.1126/science.aaj2239 PMC8009048 28473536

[50] Lazarovici A (2013). Probing DNA shape and methylation state on a genomic scale with DNase I. Proc. Natl. Acad. Sci. USA.

[51] Jaffe AE (2016). Mapping DNA methylation across development, genotype and schizophrenia in the human frontal cortex. Nat. Neurosci..

[52] Shi J (2014). Characterizing the genetic basis of methylome diversity in histologically normal human lung tissue. Nat. Commun..

[53] Bell CG (2016). Novel regional age-associated DNA methylation changes within human common disease-associated loci. Genome Biol..

[54] Sudmant PH (2015). Global diversity, population stratification, and selection of human copy-number variation. Science.

[55] Yanez-Cuna JO (2014). Dissection of thousands of cell type-specific enhancers identifies dinucleotide repeat motifs as general enhancer features. Genome Res..

[56] Gymrek M (2016). Abundant contribution of short tandem repeats to gene expression variation in humans. Nat. Genet..

[57] Ward MC (2012). Latent regulatory potential of human-specific repetitive elements. Mol. Cell.

[58] Grandi FC (2015). Retrotransposition creates sloping shores: a graded influence of hypomethylated CpG islands on flanking CpG sites. Genome Res..

[59] Speed D, Cai N, Johnson MR, Nejentsev S, Balding DJ (2017). Reevaluation of SNP heritability in complex human traits. Nat. Genet..

[60] Ragvin A (2010). Long-range gene regulation links genomic type 2 diabetes and obesity risk regions to HHEX, SOX4, and IRX3. Proc. Natl. Acad. Sci. USA.

[61] Yang J (2012). FTO genotype is associated with phenotypic variability of body mass index. Nature.

[62] Paul DS (2016). Increased DNA methylation variability in type 1 diabetes across three immune effector cell types. Nat. Commun..

[63] Andrews SV, Ladd-Acosta C, Feinberg AP, Hansen KD, Fallin MD (2016). “Gap hunting” to characterize clustered probe signals in Illumina methylation array data. Epigenetics Chromatin.

[64] Feber A (2014). Using high-density DNA methylation arrays to profile copy number alterations. Genome Biol..

[65] Cohen, J. *Statistical Power Analysis for the Behavioral Sciences*, 2nd edn (L. Erlbaum Associates, 1988).

[66] Chavez L (2010). Computational analysis of genome-wide DNA methylation during the differentiation of human embryonic stem cells along the endodermal lineage. Genome Res..

[67] Pruim RJ (2010). LocusZoom: regional visualization of genome-wide association scan results. Bioinformatics.

[68] Zarrei M, MacDonald JR, Merico D, Scherer SW (2015). A copy number variation map of the human genome. Nat. Rev. Genet..

[69] Willems T, Gymrek M, Highnam G, Mittelman D, Erlich Y (2014). The landscape of human STR variation. Genome Res..

[70] Gymrek M (2016). Abundant contribution of short tandem repeats to gene expression variation in humans. Nat. Genet..

[71] Halachev K, Bast H, Albrecht F, Lengauer T, Bock C (2012). EpiExplorer: live exploration and global analysis of large epigenomic datasets. Genome Biol..

[72] Andersson R (2014). An atlas of active enhancers across human cell types and tissues. Nature.

[73] Quinlan AR, Hall IM (2010). BEDTools: a flexible suite of utilities for comparing genomic features. Bioinformatics.

[74] McLean CY (2010). GREAT improves functional interpretation of cis-regulatory regions. Nat. Biotechnol..

[75] Houseman EA (2012). DNA methylation arrays as surrogate measures of cell mixture distribution. BMC Bioinformatics.

[76] Teschendorff AE (2013). A beta-mixture quantile normalization method for correcting probe design bias in Illumina Infinium 450 k DNA methylation data. Bioinformatics.

