# Supplementary material for: Obligatory and facilitative allelic variation in the DNA methylome within common disease-associated loci
Source: Nat Commun. 2018 Jan 2;9:8. doi: 10.1038/s41467-017-01586-1 (PMC5750212; doi:10.1038/s41467-017-01586-1)
Supplement: Supplementary file 1 — Supplementary Information [file 41467_2017_1586_MOESM1_ESM.pdf]

A

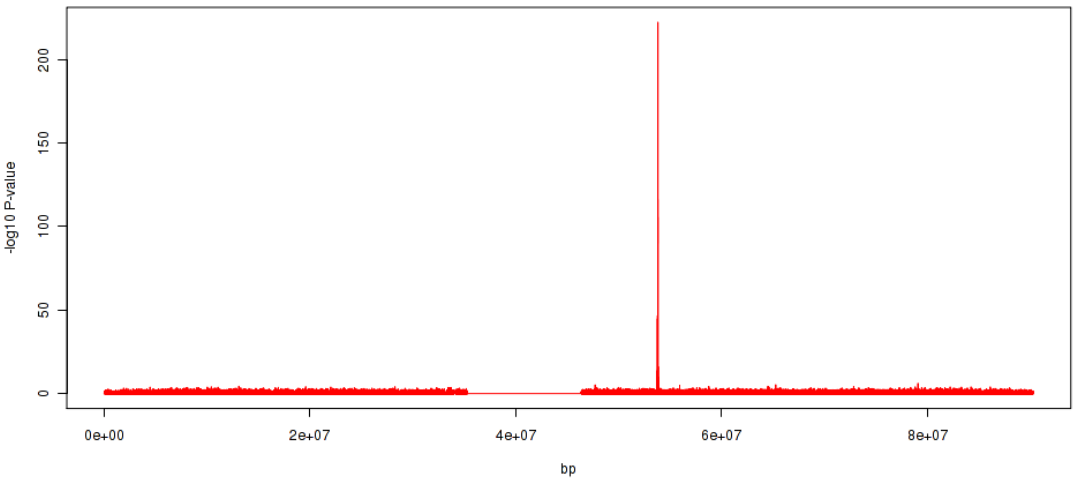

Chromosome 16

B

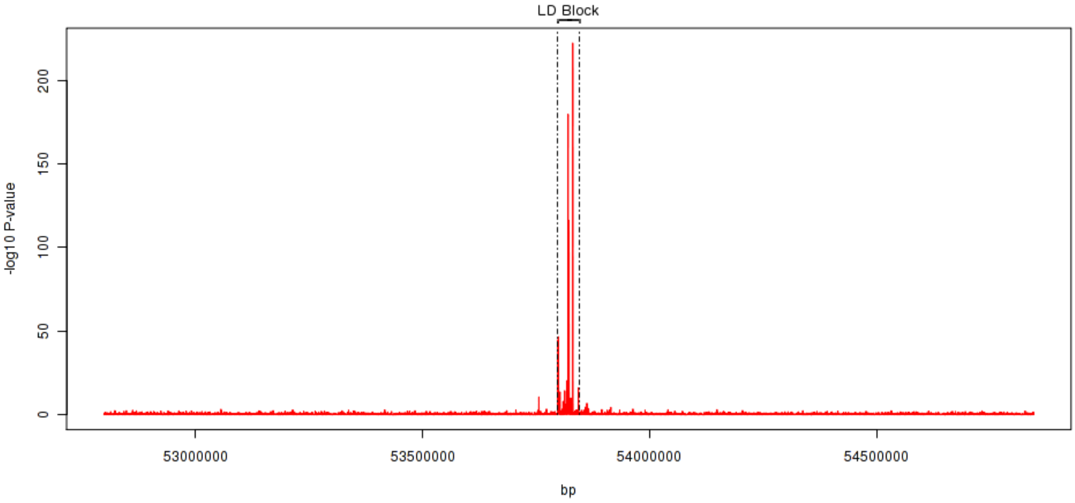

Supplementary Figure 1: Haplotype-specific methylation Analysis for SNP rs8050136 within the *FTO* LD block.

(A) Results for the entire chromosome and (B) a close up of a 2Mb window indicating Linkage Disequilibrium block that SNP resides in.

The use of recombination-derived LD Blocks dramatically reduces the regions analysed per GWAS SNP and therefore significantly increases our power to identify robust differences that are consistent across all 3 datasets. This benefit can be illustrated for the results of GWAS SNP rs8050136 within the *FTO* locus, which are concentrated within the LD Block, when compared to results derived across (A) the whole chromosome and (B) a close-up view within a ~2Mb region. Extension to whole genome would severely impact on our significance thresholds and disproportionally include non-associated regions. Also an arbitrary physical cut off distance would be unsuitable across different GWAS SNPs.

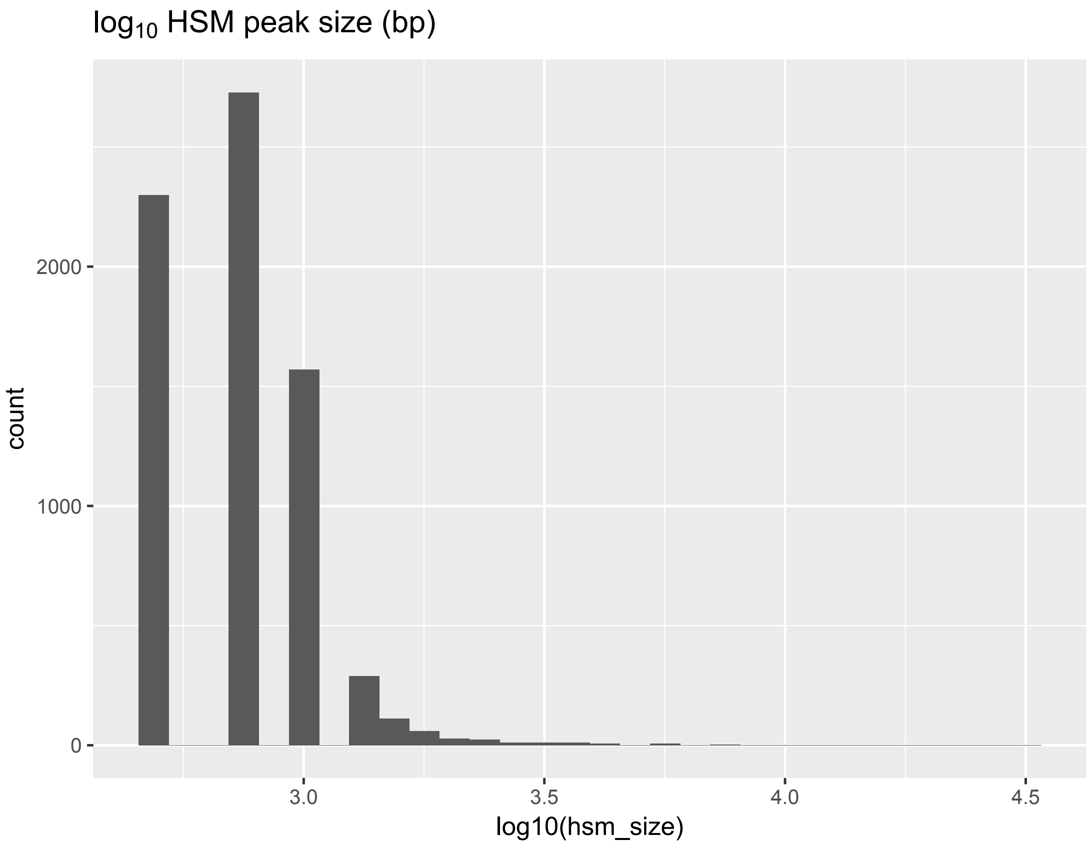

Supplementary Figure 2: HSM Peak Size
HSM peak size in log_{10} bp.

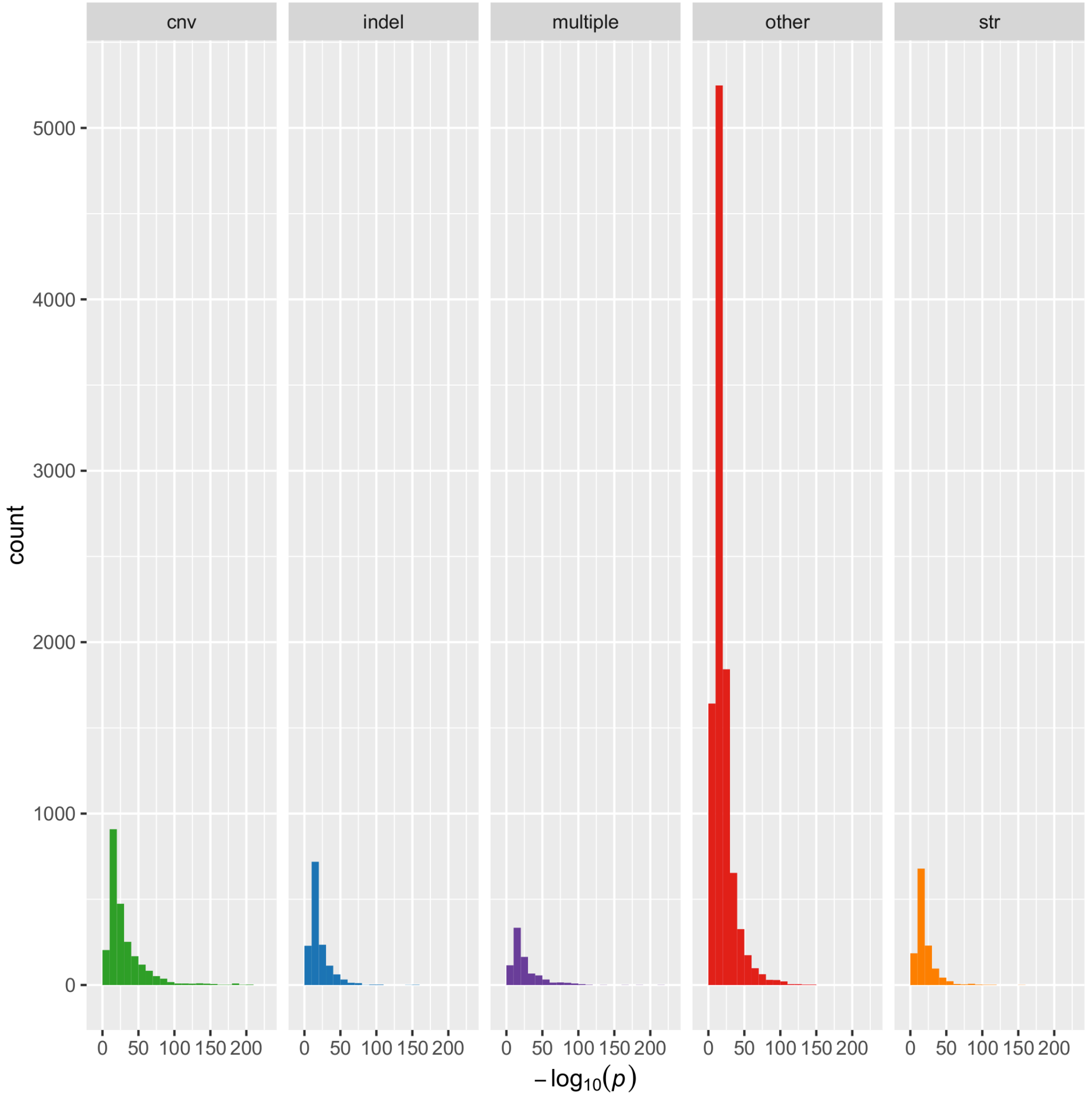

Supplementary Figure 3: HSM peak p-value distribution by Variant classes

HSM peak p-value distribution histogram for the major variant classes of the Bonferroni significant windows: CNV (Copy Number Variants); Indels (Insertion-Deletions); Multiple Variants; Other; and STR (Short Tandem Repeats). All variant categories co-locate with HSM peak locations, however the largest contribution is from the 'Other' category comprising strong influence from CpG-SNPs.

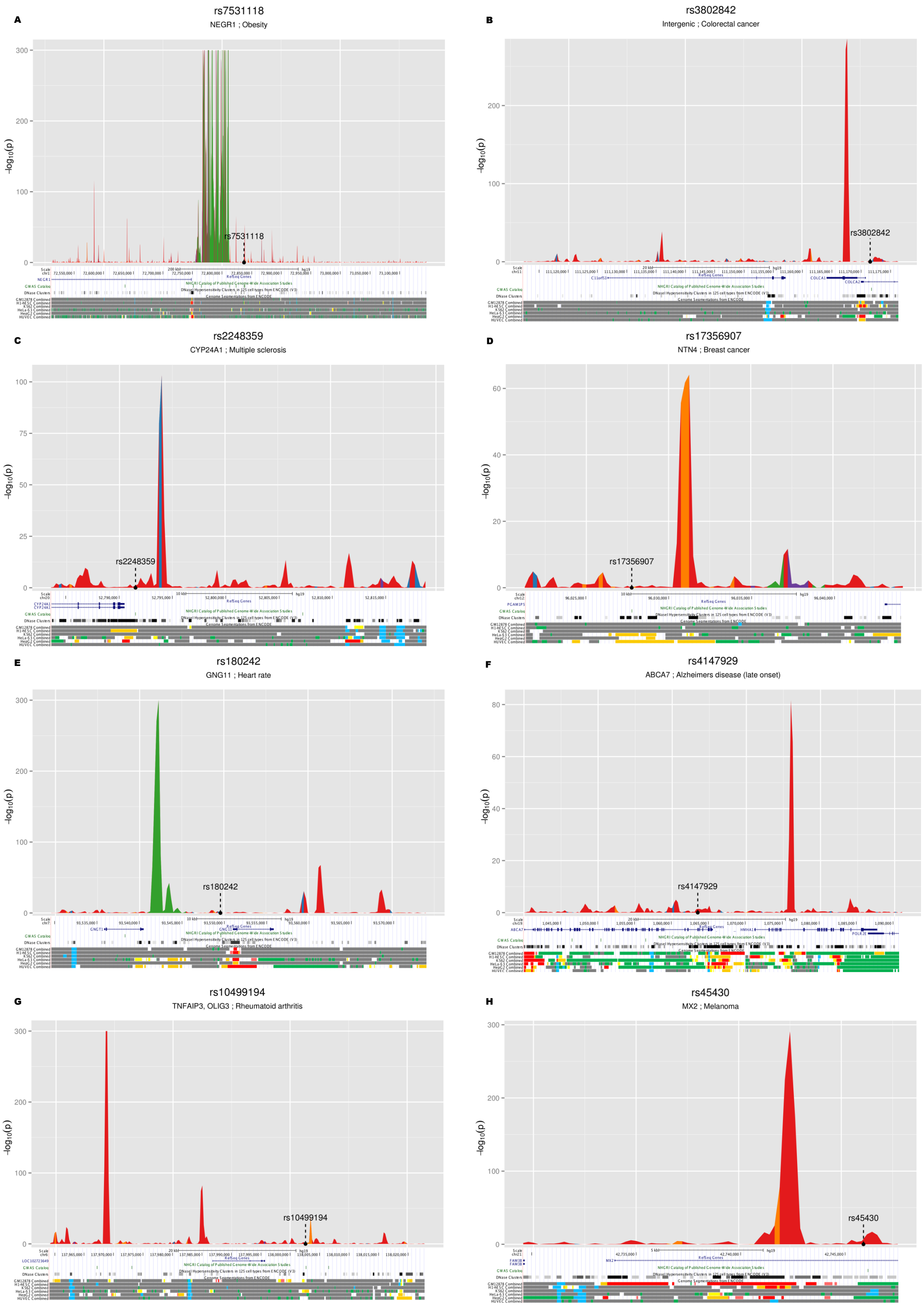

Supplementary 4: HSM Analysis for Entire Dataset

HSM Analysis for identical regions as Figure 3 in combined set for all 3,128 samples for DNA methylation with respect to GWAS SNP allelic count. This mirrors precisely the Figure 3 results but with increased power. The complete combined result had consistent overlap with the HSM peak set (7,163 of the 7,173; 99.86%).

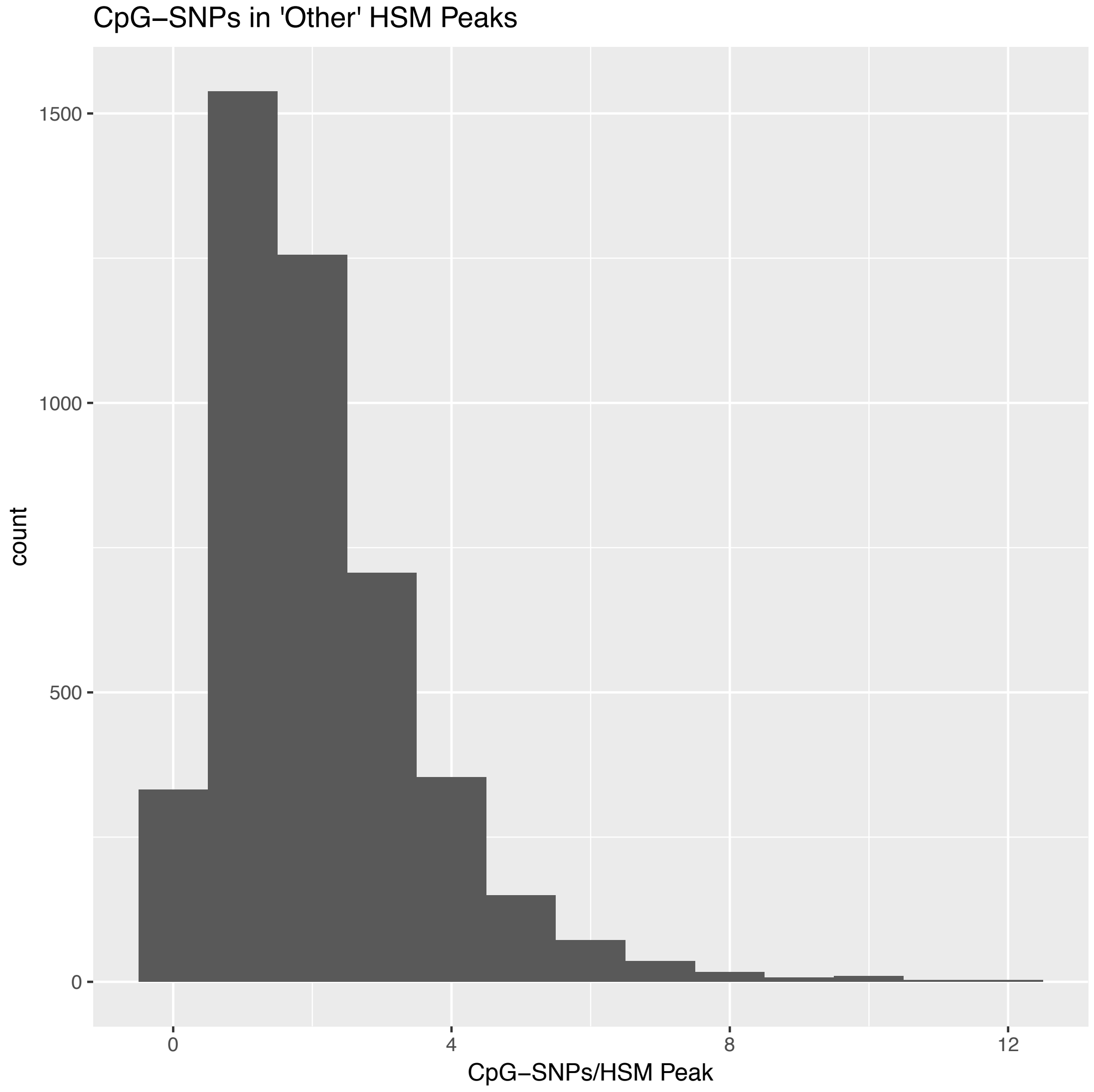

Supplementary Figure 5: CpG-SNPs within ‘Other’ variant HSM peaks

CpG-SNPs clusters contribute to a majority, but not all, HSM peaks within the ‘Other’ category, *i.e.* HSM peaks that do not overlap CNVs, Indels or STRs, or combinations of these.

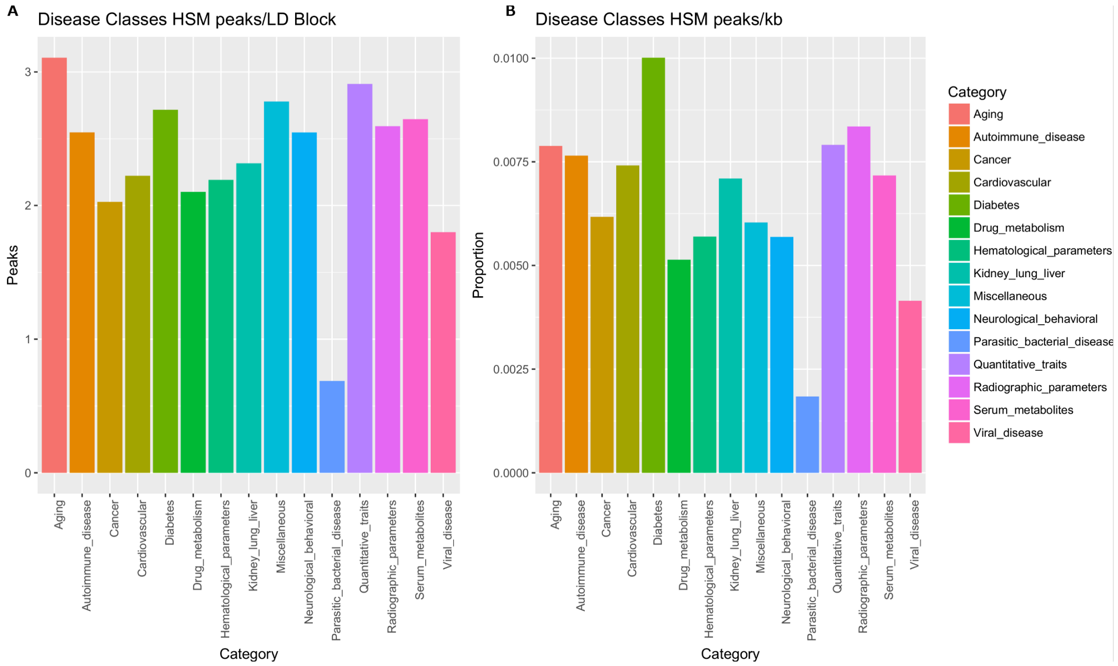

Supplementary Figure 6: HSM Peaks in LD Blocks by Disease Class

GWAS SNP results were classified into 15 groups as by Maurano *et al.*[1] (Aging; Autoimmune disease; Cancer; Cardiovascular; Diabetes; Drug metabolism; Haematological parameters; Kidney, lung and liver; Miscellaneous; Neurological and behavioural; Parasitic and bacterial disease; Quantitative traits; Radiographic parameters; Serum metabolites; and Viral disease). The number of HSM peaks per each GWAS LD Block association (A) and corrected for any size bias (B) by calculating HSM peaks/kb do not reveal any significantly difference for more blood-related diseases.

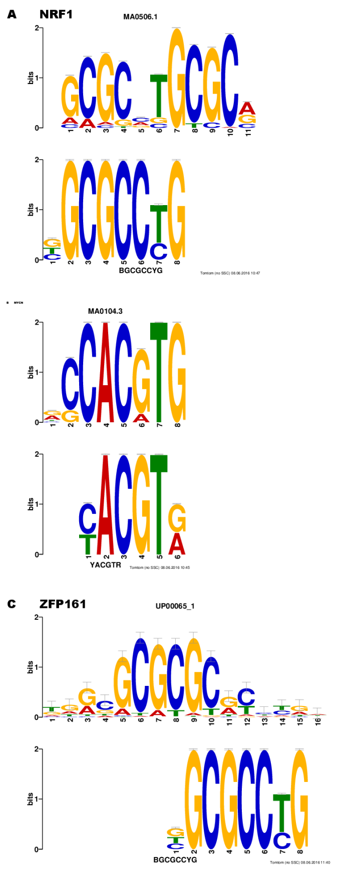

Supplementary Figure 7. Transcription Factor Motif Enrichment in HSM Peaks
Results of 3 of the Transcription factor moitf enrichments identified by MEME-suite/TOMTOM. In total 19 enriched sequences were matched which included the motifs for A) NRF1, B) MYCN, and C) ZFP161.

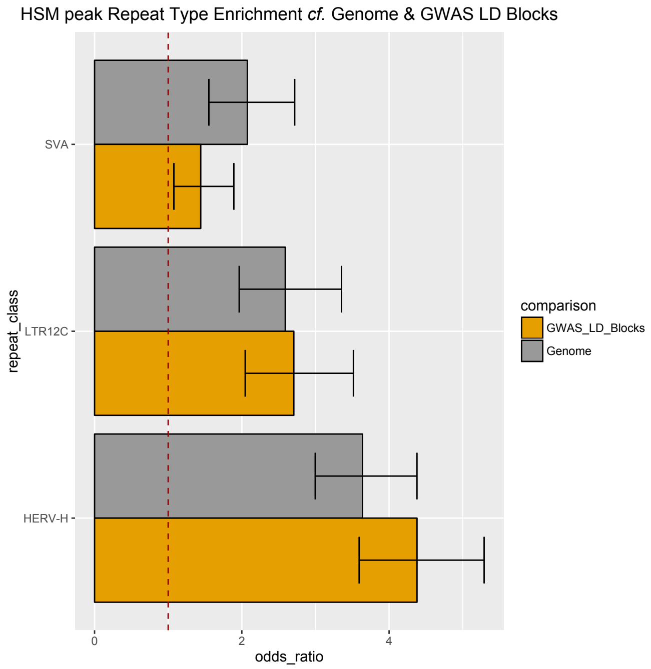

Supplementary Figure 8. HSM Peak Repeat Enrichments

SVA, LTR12C and HERV-H repeat class enrichment in HSM peaks compared to the genome and GWAS LD Block Regions. Odds Ratio (OR) from Fisher's Exact Test is shown within both comparisons to Genome (Grey) and the GWAS LD Block Region (Gold) as background. Error bars indicate the 95% Confidence Intervals, dotted red line is OR = 1.

Zero results across all samples by Functional Unit (Average)	
Genome	6.92%
GWAS LD blocks	4.07%
Genecode protein-coding gene transcripts	0.61%
Functional regions defined by Combined Segmentation within the blood-derived GM12878	0.09%
Promoter Flanking	0.03%
Transcription Start Site	0.02%
CTCF	0.07%
Weak Enhancer	0.02%
Enhancer	0.01%
Transcribed Regions	0.07%
Repressed	0.13%

Supplementary Table 1. MeDIP-seq coverage across Functional Annotations

MeDIP-seq coverage differences were compared via the proportion of zero coverage 500 bp windows across the differing Combined Chromatin Segmentation analysis functional units. This indicated no significant influence on our enrichment calculations.

Supplementary Note 1

cis-Regulatory Region Enrichment Analysis

Using the Genomic Regions Enrichment of Annotations Tool (GREAT 3.0.0[2]), we analysed the HSM peaks for gene ontology and *cis*-regulatory enrichment. Compared to the genomic background, these loci were strongly enriched for a large number of biological processes, human phenotypes and diseases ontologies (all Binominal FDR Q-value < 0.05, Supplementary Data 6). However, when comparing these loci against a background set of the GWAS LD block Regions they were not significantly more enriched for function, reflecting their general equivalence within these GWAS regions. Thus, suggesting that any enrichment of HSM peaks for diseases and categories of ontology descriptors is derived through the HSM peaks residing with the associated GWAS LD Block Regions.

Supplementary Note 2

Tissue-independent effects in multiple disease type blocks

DNA methylation differences that are strongly genetically driven, as identified in this analysis, will result in obligatory changes across multiple tissues types. As such these tissue-independent changes may be contributing towards susceptibility to human traits within these loci across a range of different organ systems, and not be as cell-type restricted as many other GWAS signals. Using the 15 broad disease classes[1], we identified 437 GWAS LD blocks that possessed HSM peaks and contain associated SNPs for more than one disease category. These results may make a thought-provoking subset for future investigation (Supplementary Data 7).

SUPPLEMENTARY REFERENCES

1. Maurano, M.T. *et al.* Systematic localization of common disease-associated variation in regulatory DNA. *Science* **337**, 1190-5 (2012).
2. McLean, C.Y. *et al.* GREAT improves functional interpretation of cis-regulatory regions. *Nature biotechnology* **28**, 495-501 (2010).
