# Supplementary material for: Obligatory and facilitative allelic variation in the DNA methylome within common disease-associated loci
Source: Nat Commun. 2018 Jan 2;9:8. doi: 10.1038/s41467-017-01586-1 (PMC5750212; doi:10.1038/s41467-017-01586-1)
Supplement: Supplementary file 3 — Description of Additional Supplementary Files [file 41467_2017_1586_MOESM3_ESM.pdf]

Description of Additional Supplementary Files

File Name: Supplementary Data 1
Description: BED file with UCSC header of 7173 HSM peak regions (GRCh37/hg19) Excel files.

File Name: Supplementary Data 2
Description: Enrichment analysis of HSM peaks across Chromatin Segmentation Annotations from ENCODE in 6 Tissues.

File Name: Supplementary Data 3
Description: Enrichment analysis of HSM peaks across Functional Categories.

File Name: Supplementary Data 4
Description: Enrichment analysis of HSM peaks compared with DNase-I Hypersensitivity Sites produced from 125 different tissue types (ENCODE).

File Name: Supplementary Data 5
Description: Enriched Transcription Factor Binding Site (TFBS) motifs via MEME-ChIP and the TOMTOM algorithm within the HSM peak DNA sequences.

File Name: Supplementary Data 6
Description: Genomic Regions Enrichment of Annotations Tool (GREAT 3.0.0) analysis of HSM peaks for gene ontology and cis-regulatory enrichment.

File Name: Supplementary Data 7
Description: GWAS LD blocks with HSM peaks associated with multiple disease categories.
