# Supplementary material for: Obligatory and facilitative allelic variation in the DNA methylome within common disease-associated loci
Source: Nat Commun. 2018 Jan 2;9:8. doi: 10.1038/s41467-017-01586-1 (PMC5750212; doi:10.1038/s41467-017-01586-1)
Supplement: Supplementary file 2 — Peer Review File [file 41467_2017_1586_MOESM2_ESM.pdf]

Reviewers' comments:

Reviewer #1 (Remarks to the Author):

This paper presents data identifying regions of blood-derived methylation variation that are associated with genetic variation at GWAS-identified SNPs. Because the analyses are limited to GWAS SNP-defined LD blocks for the genetic variation and 500bp windows within that region for methylation variation, they term this "haplotype specific methylation – HSM". They identify blood-based HSM lists for ~8000 GWAS SNP hits - after removing duplicated phenotypes and LD overlap, there were ~2700 unique GWA S LD Blocks considered in HSM analyses.
Upon identification of HSMs, the authors then explore enrichment of these HSMs for multiple factors, comparing to the full genome, and to all GWAS LD Blocks. These factors included non-SNP sequence variation, chromatin features from ENCODE data on 6 tissues, functional annotations (e.g. DNAse Hypersenstivity sites, TSSs, etc), CG density, and CpG-SNPs (SNPs at a CpG involved in methylation). They conclude that the enrichment of particular features among these HSMs for known GWAS hits is important new information that can guide further functional follow up of GWAS hits.
While I think this paper reflects a lot of work that should be presented to peers, and thus merits publication in some form, I have several hesitations about the clarity of the presentation and specific conclusions made:
1. The primary analysis is identifying the HSMs from GWAS LD blocks. This is very similar to mQTL analyses reported by several groups, with a few modifications: (a) considers clustered methylation rather than single CpGs; (b) focuses on the methylation "target" rather than on the controlling SNP (mQTL); (c) limits analysis to GWAS hit regions rather than whole genome. This similarity to mQTL work is not mentioned in the introduction or early results. I think it should be to orient readers to the similarity and differences from other work.
2. The HSM list that sets the stage for all the enrichment analyses that follow is based on blood-derived methylation data. This is not necessarily a weakness, as this is the accessible tissue from which much of the EWAS field has been derived, but it is important to mention early on and to refer back to often. How different would such results be for other tissue types? Should the GWAS hit list have been restricted only to phenotypes where blood is a feasibly functional tissue? If not, as was done here, why not?
3. The non-SNP variant enrichment analysis shows that repeat and copy number variation are enriched in HSMs vs GWAS LD blocks, with the comment "as expected". I agree with this expectation - HSMs are detectible as differences in methylation intensity – a feature related to this type of variation. Since this was already expected, I am having trouble understanding what the authors intending point out in this analysis (i.e. fig 2). Figures 3 and 4 are on the same theme and point to the "other" category of variants as being a major contributor to HSM findings compared to CNVs, indels, etc. This does not seem to be the stated point in the text for that section, however, so not sure I have interpreted correctly. My suggestion would be to reduce figs 2 – 4 into only the critical result intended to be highlighted and then state that result clearly and succinctly in that section. The point in this section is related to results in fig 8 as well. Would it be helpful to put these all together in context?
4. It appears that HSMs had to be subsets of locations within the GWAS LD block. Presumably this is to help further localize the region to functional pieces. However, there are a few downsides to limiting analysis to subsets bounded by the LD block location. First, it does not allow for methylation associations outside the LD block defined for a GWAS SNP. This means that new regions will not be implicated by this, which, as others are starting to show, is certainly a possible scenario that would greatly add functional insights beyond what the GWAS hit implies. Second, because the genomic region boundary is already set by the LD block from GWAS, couldn't all the enrichment work simply have been done comparing those to the genome? What does the HSM layer add specifically? The enrichment analyses compared HSM to LD blocks, and HSM to genome, but not LD blocks to genome (as GWAS papers have done). A useful comparison would be to see how much new insight is achieved via the analyses presented here and analyses that would just use GWAS data (LD block to genome).

5. Results related to fig 7 indicate depletion of CGIs in HSMs. This seems to me expected given the design of the experiment using MeDIP-seq. Can the authors expand on what their expectation was a priori? It would also be very useful to know what genome-coverage was obtained using meDIP-seq (in terms of fraction of genes, fraction of GWAS LD blocks, etc; not number of sequencing reads). The enrichment results are difficult to interpret without knowing whether these regions were represented via the methods used.
6. The discussion section makes several comments that are hard to follow regarding what the authors intend as “take-away” messages. The third paragraph of discussion highlights the utility of examining methylation correlates of genetic sequence, and thus not conditioning out mQTL targets when doing EWAS. The fifth paragraph also seems to say that removal of genetic signal for EWAS is a bad idea. It goes on to cite multiple references about overlap of CpG-SNPs and HSMs and other functional factors. It is not quite clear what the point of that paragraph is however. Then, the last paragraph of the discussion seems to argue that genetic “confounding” cannot be underestimated, implying a cautionary tail for EWAS and that such adjustment should be done. Put together, this section is, to me, very unclear about the interpretation and recommendations being made by the authors. What is the suggestion concretely, based on the data presented?
7. Similarly, the overall conclusions intended by the authors are not clear to me. The final “conclusion” paragraph I think simply intends to state that there are regions of methylation associated with genetic haplotypes know to be associated with disease/phenotypes from GWAS, and that these are functionally enriched, so identifying them and their functional correlates is an important step in the GWAS hit follow-up process. Is that the intended full conclusion? Why bring up more jargon like hepitypes?
8. The writing style of this paper is difficult to consume throughout. Complicated language is used where simple language would suffice, making the facts of what was actually done in the analysis difficult to follow as well as the intended conclusions. There are also words missing or mistyped (e.g. were instead of where) in multiple places.

Reviewer #2 (Remarks to the Author):

This paper is dedicated to the better understanding of DNA – epigenetic interactions and their roles in human diseases as well as new interpretations of GWAS signals. More specifically, the authors investigated DNA methylation variation and differences between risk and non-risk haplotypes. They mapped the peaks of haplotype specific methylation using MeDIP-seq data from a large blood-based sample which was also genotyped on SNP arrays.

This work represents an extension of the first author’s effort to identify T2D and obesity risk factors using genetic and DNA methylation information of the FTO gene (PLOS One 2010). In the current submission, the main methodological aspects as well as technical standards are very similar to the ones described in the 2010 paper.

In the 2nd paragraph of the manuscript the authors categorized DNA-epigenetic interactions into 3 groups: pure, facilitated, and obligatory (when sequence variants directly predict the epigenetic state). In my opinion, the main problem with this study was the lack of clear separation of the three groups. The genetically heterogeneous population cannot be used for identification of the first group, so only the latter two are left. From reading the manuscript, I got an impression is that CNVs and CpG-SNPs played a key role in haplotype differences which initially were treated as methylation differences. But this would be a misnomer for evident reasons. In the case of CNV, DNA dosage effects can simulate epigenetic differences. In the case of CG-> (non-C)G polymorphism/mutation, the C substitute would eliminate the target of DNA methylation, and therefore prohibit MeDIP enrichment of this allele. As an outcome of the failure differentiate, obligatory interactions from the facilitated ones does not allow to estimate the additive value of epigenetic factors in mapping disease-relevant functional parts of risk DNA haplotypes.

It would be helpful to have more information on the HSM peaks. How long are they? Fig 4 illustrates such peaks but the font is so small that it is not possible to get any useful information from them. If the peaks are relatively short, e.g. within a single window of ~500 bp, do they still represent haplotypic effects? Or they are cases of meQTL or allele-specific methylation? In this context, some relevant papers which investigated meQTL in pan-GWAS context, e.g. Jaffe et al. Nature Neuroscience 2015 (or 2016), should be cited and discussed.

We thank the reviewers very much for their time and for their detailed and insightful comments. We have made a point-by-point response to all these questions raised below, which we believe answer these queries robustly and have led to significant improvements in the manuscript.

Reviewer #1 (Remarks to the Author):

This paper presents data identifying regions of blood-derived methylation variation that are associated with genetic variation at GWAS-identified SNPs. Because the analyses are limited to GWAS SNP-defined LD blocks for the genetic variation and 500bp windows within that region for methylation variation, they term this "haplotype specific methylation – HSM". They identify blood-based HSM lists for ~8000 GWAS SNP hits - after removing duplicated phenotypes and LD overlap, there were ~2700 unique GWAS LD Blocks considered in HSM analyses.
Upon identification of HSMs, the authors then explore enrichment of these HSMs for multiple factors, comparing to the full genome, and to all GWAS LD Blocks. These factors included non-SNP sequence variation, chromatin features from ENCODE data on 6 tissues, functional annotations (e.g. DNase Hypersensitivity sites, TSSs, etc.), CG density, and CpG-SNPs (SNPs at a CpG involved in methylation). They conclude that the enrichment of particular features among these HSMs for known GWAS hits is important new information that can guide further functional follow up of GWAS hits.
While I think this paper reflects a lot of work that should be presented to peers, and thus merits publication in some form, I have several hesitations about the clarity of the presentation and specific conclusions made.

We thank the reviewer for their positive comments on our manuscript and that this analysis is worthy of publication.

1. The primary analysis is identifying the HSMs from GWAS LD blocks. This is very similar to mQTL analyses reported by several groups, with a few modifications: (a) considers clustered methylation rather than single CpGs; (b) focuses on the methylation "target" rather than on the controlling SNP (mQTL); (c) limits analysis to GWAS hit regions rather than whole genome. This similarity to mQTL work is not mentioned in the introduction or early results. I think it should be to orient readers to the similarity and differences from other work.

   Yes we definitely agree with reviewer that similarity and differences to previous mQTL analysis should be included in the manuscript. The following sentences have now been included at the beginning of the Results section.

   "Our analysis is similar in concept but methodological different to previous DNA methylation quantitative trait loci (mQTL) analyses performed to identify significant SNP associations with DNA methylation levels via array data[1-3]. We

focus specifically on those genetic changes already robustly associated with disease via GWAS and additionally, the use of MeDIP-seq data enables the entire related LD block regions to be interrogated, not isolated and predetermined CpGs. Whilst only regional DNA methylation changes can be identified via these data, where they can be found, they are inherently more functional loci[4]."

2. The HSM list that sets the stage for all the enrichment analyses that follow is based on blood-derived methylation data. This is not necessarily a weakness, as this is the accessible tissue from which much of the EWAS field has been derived, but it is important to mention early on and to refer back to often. How different would such results be for other tissue types? Should the GWAS hit list have been restricted only to phenotypes where blood is a feasibly functional tissue? If not, as was done here, why not?

   Yes we agree with the reviewer that it should be clearly stated to readers of the manuscript that these are peripheral blood-derived results. We have modified the text in the Introduction, Results, and Methods to again stress this, as detailed below.

   Introduction:
   "We used genome-wide Methylation-Dependent Immunoprecipitation 2nd-generation sequencing (MeDIP-seq) data derived from peripheral blood and high-quality SNP array genotype data from 3,128 samples of European ancestry."

   Results:
   "The total peripheral blood derived DNA methylome samples were split into three separate datasets (Figure 1 and Methods)."

   Methods:
   "For this study, peripheral blood derived DNA was provided by the adult volunteers from the UK Twin Register (TwinsUK Resource)"

   Data from our analysis supports the assumption that the HSM peaks we have identified are strongly driven, particularly, by obligatory genetic effects, which will be observed across all tissues. This includes the results from the cell-type specific DNase-I Hypersensitivity site data overlap with HSM peaks. These results do not show any bias towards the 22 blood derived tissues from the total 125 tissue types (Figure R1 below and Figure 8).

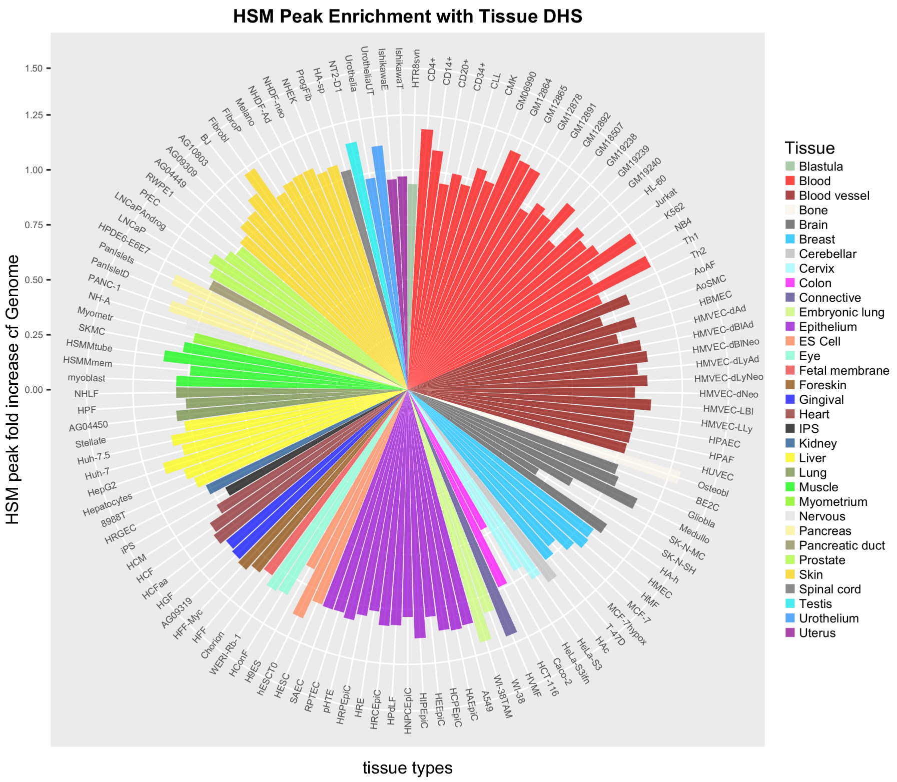

Figure R1 (and Figure 8 in paper). HSM peaks overlapped with 125 tissue DNase-I Hypersensitivity sites via ENCODE.

Additionally, as suggested by the reviewer, we have further explored the potential tissue-specific enrichment of our results. We examined the HSM peaks with respect to their GWAS SNP associations classified by the 15 broad disease categories determined by Maurano *et al.*[5]. Calculated as either number of HSM peaks per GWAS SNP LD block, or size-adjusted HSM peaks/kb, this analysis does not reveal any significant evidence for a bias towards blood-related disease groupings, such as autoimmune, cardiovascular, hematological parameters or serum metabolites, in comparison to the other categories (See Figure R2A & R2B and new Supplementary Figure S6). The only significant outlier is the low results for parasitic-related associations, but this is likely due to this grouping only containing 16 associations, by far the smallest number. We have also added this analysis to our results section as detailed below:

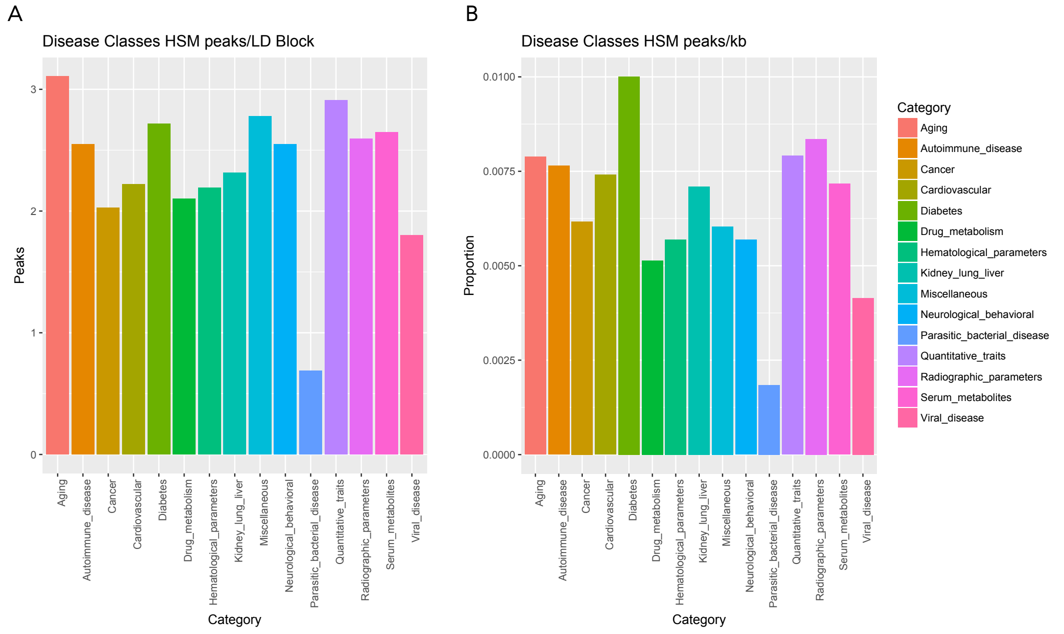

Figure R2: GWAS SNP results were classified into 15 groups as by Maurano *et al*. (Aging; Autoimmune disease; Cancer; Cardiovascular; Diabetes; Drug metabolism; Haematological parameters; Kidney, lung and liver; Miscellaneous; Neurological and behavioural; Parasitic and bacterial disease; Quantitative traits; Radiographic parameters; Serum metabolites; and Viral disease). (A) The number of HSM peaks per each GWAS LD Block association and (B) corrected for any size bias by calculating HSM peaks/kb do not reveal any significantly difference for more blood-related diseases (also Supplementary Figure S6).

Results:

"Additionally, we further explored for any evidence that our results are biased towards blood-related disorders using the 15 broad disease categories for GWAS associations as classified by Maurano *et al*.[5]. We see no enrichment for HSM peaks to reside within GWAS LD blocks of blood-specific or related diseases above any of the other categories, either by number of HSM peaks per GWAS LD Block or size corrected HSM peaks/kb (see Supplementary Figure S6)"

3. The non-SNP variant enrichment analysis shows that repeat and copy number variation are enriched in HSMs vs GWAS LD blocks, with the comment "as expected". I agree with this expectation - HSMs are detectible as differences in methylation intensity – a feature related to this type of variation. Since this was already expected, I am having trouble understanding what the authors intending point out in this analysis (i.e. fig 2). Figures 3 and 4 are on the same theme and point to the "other" category of variants as being a major contributor to HSM findings compared to CNVs, indels, etc. This does not seem to be the stated point in the text for that section, however, so not sure I have interpreted correctly. My suggestion would be to reduce figs 2 – 4 into only the critical result

intended to be highlighted and then state that result clearly and succinctly in that section. The point in this section is related to results in fig 8 as well. Would it be helpful to put these all together in context?

Yes we agree with the reviewer. The major point we wish to convey is that whilst effects related to CNVs *etc.* are significant contributors and may led to significant regional or neighbouring effects, they do not explain all of these strong genetically-associated differences. In fact, the 'Other' category includes 63.2% of the HSM peaks. To further stress this we have modified the text in the Abstract, Introduction and Results as detailed below.

Abstract:

"Our results demonstrate strong allelic differences in DNA methylation driven by obligatory or facilitative genetic effects, that may have direct or regional functional impact in these common disease-related regions."

Introduction:
"This leads to a distinct signal of population variation in both facilitated and obligatory genetic effects on the DNA methylome."

Results:
"Thus whilst large scale variants, such as CNVs, do have an increased impact within these regions, they do not account for all the signal with 63.2% apportioned to the 'Other' category."

We have also as suggested removed Figure 3 to the Supplementary Section and moved the discussion of CpG and CpG-SNP density to this part of the manuscript, so Figure 8 now becomes Figure 5. However, we have retained Figure 3 as this displays some representative results with these GWAS LD Block regions to orientate the reader. Furthermore, all these graphs and results can be now be browsed and downloaded for respective GWAS association SNPs, disease-related regions or the entire dataset at http://epigenome.soton.ac.uk/hsm/hsm.php

4. It appears that HSMs had to be subsets of locations within the GWAS LD block. Presumably this is to help further localize the region to functional pieces. However, there are a few downsides to limiting analysis to subsets bounded by the LD block location.
   a. First, it does not allow for methylation associations outside the LD block defined for a GWAS SNP. This means that new regions will not be implicated by this, which, as others are starting to show, is certainly a possible scenario that would greatly add functional insights beyond what

the GWAS hit implies.

Yes we agree with the reviewer that interesting DNA methylation associations can be found beyond the boundaries of the GWAS LD blocks. However, our focus was to identify direct obligatory and potentially facilitated genetic effects on the DNA methylome strongly correlated with these GWAS SNPs. Using these recombination defined cut-offs dramatically increases our power, by reducing our multiple testing correction. We are excluding large regions of non-significant windows beyond these boundaries and/or the use of an arbitrary physical size defined boundaries that would be inappropriate across different LD blocks. Performing this analysis for the whole genome for each GWAS SNP would have reduced our significance level by a magnitude of 5.

For example, a chromosome 16 wide analysis of the rs8050136 SNP (*FTO*) (Figure R3A) and a more focused ~2 Mb region (Figure R3B), shows that the robust results we are finding are strongly concentrated within the recombination-defined GWAS LD block regions.

Specifically hunting for pure, non-genetically driven, epigenetic changes impacted upon by beyond these GWAS LD Block regions would be an interesting analysis, although dramatically less powerful even in this large dataset, and is beyond the focus of this current study. We now include these figures as Supplementary Figures S1A and S1B and include further discussion of this point within the methods section.

Results:

"We focus only on those genetic changes already robustly associated with disease via GWAS and, additionally, the use of MeDIP-seq data enables the entire related LD block regions to be interrogated, not isolated and predetermined CpGs. To identify obligatory or facilitated genetic effects on DNA methylation that are in strong LD with the GWAS SNP, we apply our analysis within the recombination-defined LD blocks, were these signals strongly reside (see Supplementary Figure S1). Whilst pure epigenetic changes may exist beyond these limits, our focused approach dramatically increases our study power to find these genetically-driven variations."

A

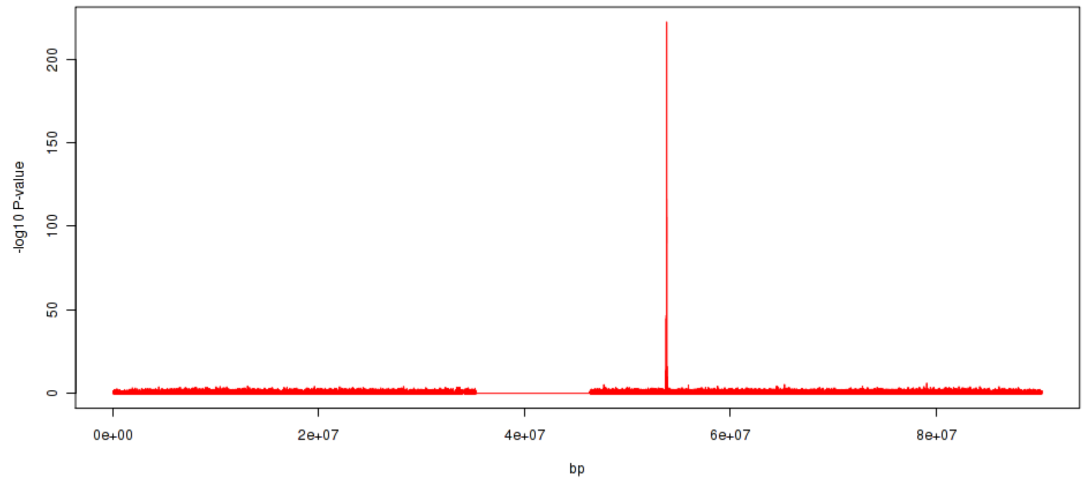

Chromosome 16

B

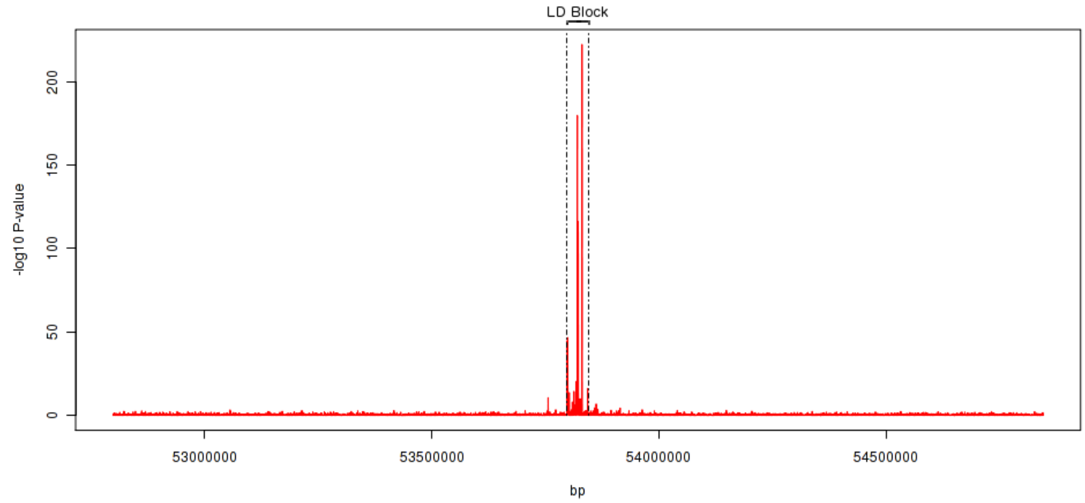

Figure R3 and Supplementary Figure S1 (A) Haplotype-specific methylation Analysis for SNP rs8050136, which resides within the *FTO* LD block. (A) Results for the entire chromosome and (B) a close up of a 2Mb window indicating Linkage Disequilibrium block that SNP resides in.

The use of recombination-derived LD Blocks dramatically reduces the regions analysed per GWAS SNP and therefore significantly increases our power to identify robust differences that are consistent across all 3 datasets. This benefit can be illustrated for the results of GWAS SNP rs8050136 within the *FTO* locus, which are concentrated within the LD Block, when compared to results derived across the whole chromosome (A) and a close-up view within a ~2Mb region (B). Extension to whole genome would severely impact on our significance thresholds and disproportionally include non-associated regions. Also an arbitrary physical cut off distance would be unsuitable across different GWAS SNPs.

b. Second, because the genomic region boundary is already set by the LD block from GWAS, couldn't all the enrichment work simply have been done comparing those to the genome? What does the HSM layer add

specifically? The enrichment analyses compared HSM to LD blocks, and HSM to genome, but not LD blocks to genome (as GWAS papers have done). A useful comparison would be to see how much new insight is achieved via the analyses presented here and analyses that would just use GWAS data (LD block to genome).

The HSM peaks indicate possibly novel regions of significant DNA methylation between the risk and non-risk GWAS SNP haplotypes. As the reviewer rightly points out, many studies have identified that GWAS LD Block regions are enriched for functionality in comparison to the genome, as we have also acknowledged. Therefore, comparing the HSM blocks to the genome will show this same enrichment. This is as long as HSM blocks are representative of the GWAS LD blocks within which they reside and are not functionally depleted exceptions within these regions. Therefore, to query this possibility we specifically compare between the HSM peaks and GWAS LD blocks. Interestingly, we find that that the HSM peaks are even more enriched than the GWAS LD Blocks in some functional categories, including DNase-I Hypersensitivity sites, Transcripts and Dynamic regions.
The genome, GWAS LD blocks and HSM peak proportions for all categories tested are all available in Supplementary Table S1 and S3 for comparison and we have added the additional explanation to clarify the points raised by the reviewer in the results.

Results:
"We then compared these regions specifically against their proportion within the GWAS LD Blocks only. This was in order to test that the HSM peaks are not depleted exceptions to the well-known functional enrichment within GWAS regions."

5. Results related to fig 7 indicate depletion of CGIs in HSMs. This seems to me expected given the design of the experiment using MeDIP-seq. Can the authors expand on what their expectation was a priori? It would also be very useful to know what genome-coverage was obtained using meDIP-seq (in terms of fraction of genes, fraction of GWAS LD blocks, etc.; not number of sequencing reads). The enrichment results are difficult to interpret without knowing whether these regions were represented via the methods used.

   Yes we agree with the reviewer that more details are required. We would expect CGIs to be under-represented in total across the genome, as the vast majority are predominantly unmethylated; this being the default state for CpG dense regions.

Therefore, we would also expect less opportunity for DNA methylation to vary due to association with GWAS SNPs within these regions. This is consistent with what we observe with a stronger depletion for HSM peaks within CGI (Figure 7). However, the 32 CpG islands identified that have at least moderate overlap (f ≥ 0.1) with HSM peaks are of obvious functional interest for those particular GWAS associations. This indicates there is possible genetic variability within these CGI in LD with the GWAS SNP that is influencing DNA methylation state with potential canonical functional consequences. This for instance could include variation within an MDR motif[6] or critical CpGs[7].

Then to answer the query about genome-coverage, we calculated the regions where there were zero results across all individuals to explore coverage across functional regions. 6.92% of the entire DNA methylome dataset contains zero reads across all individuals in this analysis. Across the GWAS LD blocks this reduces to an average of 4.07% across each block. Across all genecode protein-coding gene transcripts this reduces significantly further to an average of 0.61%. Furthermore, across the functional regions defined by Combined Segmentation within the blood-derived GM12878 the average is 0.09%, with categories breaking down as follows: Promoter Flanking = 0.03%; Transcription Start Site = 0.02%; CTCF = 0.07%; Weak Enhancer = 0.02%; Enhancer = 0.01%; Transcribed Region = 0.07%; Repressed = 0.13%.

Results:

"Whilst we expect a depletion within CGI across the genome, as these regions are predominately unmethylated, the 32 CGI in which we do find GWAS-related DNA methylation variation are of obvious interest for those traits. This indicates potential genetic variability within these CGI, in LD with the GWAS SNP, which is strongly influencing DNA methylation state. This may therefore have canonical functional consequences."

We have also included the discussion of the above coverage data in the Results and an additional Supplementary Table 2.

Results:
"Generalised MeDIP-seq coverage differences were compared via the proportion of zero coverage across the differing functional units and indicated no significant influence on our enrichment calculations (See Supplementary Table 2)."

6. The discussion section makes several comments that are hard to follow regarding what the authors intend as "take-away" messages. The third paragraph of discussion highlights the utility of examining methylation correlates of genetic sequence, and thus not conditioning out mQTL targets when doing EWAS. The

fifth paragraph also seems to say that removal of genetic signal for EWAS is a bad idea. It goes on to cite multiple references about overlap of CpG-SNPs and HSMs and other functional factors. It is not quite clear what the point of that paragraph is however. Then, the last paragraph of the discussion seems to argue that genetic "confounding" cannot be underestimated, implying a cautionary tail for EWAS and that such adjustment should be done. Put together, this section is, to me, very unclear about the interpretation and recommendations being made by the authors. What is the suggestion concretely, based on the data presented?

Yes we agree with the reviewer that this section could be more clearly presented. We have rewritten the discussion shortening it considerably.
Our recommendations are:

1) To be aware of the potential genetic confounding and/or obligatory or facilitative interaction both directly and due to regional or neighbouring effects.
2) To use this robust HSM dataset to check for this potential - Supplementary File 1 and http://epigenome.soton.ac.uk/hsm/hsm.php
3) To further explore for additional evidence of strong genetic effect, such as "gap hunting" in DNA methylation data that may indicate the strongest of these genetic influences[8] as well as population-specific data availability of CpG-SNPs and larger genetic variants.
4) Furthermore, where common SNP data is also available for study cohort, the inclusion of, or investigation of top hits, by a haplotype-tagging allelic count analysis for the LD block within which the probe(s) resides may reveal unknown regional or haplotypic effects on the differential DNA methylation observed.
5) When these genetic effects can be identified then exploration of its nature, whether obligatory, facilitative, or dosage effects with potential regional ramifications is required.
6) However, we acknowledge that currently there is often no easy answer to the complex interplay between genetic influence and observed epigenetic state. Interpretation needs to be realistic and pragmatically aware that genetic *cis* or *trans* effects are not always completely excludable.
7) Technological advances, including direct assessment of DNA modifications in long reads to enable more robust genetic and epigenetic haplotypic assessment will obviously improve our knowledge of these interactions and their interplay in disease risk.

We have modified the discussion as below:

"Therefore, researchers need to be as aware as possible of the potential genetic confounding and/or interaction, both directly or due to regional or neighbouring effects. Our dataset adds to the available genetic effects to be integrated in these

analyses (Supplementary File 1). Researchers should also explore for additional evidence of strong genetic effects, such as "gap hunting" in DNA methylation data that may indicate the strongest of these genetic influences,[71] as well as population-specific data availability of CpG-SNPs and larger genetic variants. Furthermore, where common SNP data is also available for study cohort, a haplotype-tagging allelic count analysis for the LD block within which the probe(s) resides may reveal unknown regional or haplotypic effects on the observed DNA methylation. We identified ~10.0% of the 450k CpG probes within these GWAS regions may be influenced ($p < 0.05$) by their lead SNP captured haplotype background, even after excluding probes with common SNP co-location. Identified effects require exploration for evidence of obligatory, facilitative, or dosage factors."

7. Similarly, the overall conclusions intended by the authors are not clear to me. The final "conclusion" paragraph I think simply intends to state that there are regions of methylation associated with genetic haplotypes know to be associated with disease/phenotypes from GWAS, and that these are functionally enriched, so identifying them and their functional correlates is an important step in the GWAS hit follow-up process. Is that the intended full conclusion? Why bring up more jargon like hepitypes?

   Yes we agree with the reviewer that this is the message that we are intending to convey as well as the points raised above. Complete allelic integration of both genetic and epigenetic variability is required to fully understand these human disease associated regions. We have now streamlined this paragraph and included a summary sentence as below and have removed the reference to hepitypes.

   Discussion:
   "Complete allelic integration of both genetic and epigenetic variability is required to fully understand these human disease associated regions."

8. The writing style of this paper is difficult to consume throughout. Complicated language is used where simple language would suffice, making the facts of what was actually done in the analysis difficult to follow as well as the intended conclusions. There are also words missing or mistyped (e.g. were instead of where) in multiple places.

   We agree with the reviewer and have re-edited the manuscript with a fresh eye, simplifying language where possible, breaking up long sentences, and as well correcting mistyped words. The Discussion section especially has been significantly shortened and rewritten.

Reviewer #2

This paper is dedicated to the better understanding of DNA – epigenetic interactions and their roles in human diseases as well as new interpretations of GWAS signals. More specifically, the authors investigated DNA methylation variation and differences between risk and non-risk haplotypes. They mapped the peaks of haplotype specific methylation using MeDIP-seq data from a large blood-based sample, which was also genotyped on SNP arrays.

This work represents an extension of the first author's effort to identify T2D and obesity risk factors using genetic and DNA methylation information of the *FTO* gene (PLOS One 2010). In the current submission, the main methodological aspects as well as technical standards are very similar to the ones described in the 2010 paper.

In the 2nd paragraph of the manuscript the authors categorized DNA-epigenetic interactions into 3 groups: pure, facilitated, and obligatory (when sequence variants directly predict the epigenetic state). In my opinion, the main problem with this study was the lack of clear separation of the three groups. The genetically heterogeneous population cannot be used for identification of the first group, so only the latter two are left. From reading the manuscript, I got an impression is that CNVs and CpG-SNPs played a key role in haplotype differences which initially were treated as methylation differences. But this would be a misnomer for evident reasons. In the case of CNV, DNA dosage effects can simulate epigenetic differences. In the case of CG-> (non-C)G polymorphism/mutation, the C substitute would eliminate the target of DNA methylation, and therefore prohibit MeDIP enrichment of this allele. As an outcome of the failure differentiate, obligatory interactions from the facilitated ones does not allow to estimate the additive value of epigenetic factors in mapping disease-relevant functional parts of risk DNA haplotypes.

Yes we thoroughly agree with reviewer. However, the extent of these non-'pure' epigenetic effects on signal and possible confounding or functional interplay with genetic factors needs stronger acknowledgement. The potential for these to impact upon analysis needs to be conveyed to the epigenetics community, as even dosage effects can impact on array analysis. For example, 24,974 and 37,018 probes from the 450k and 850k, respectively, overlap the stringent set of CNVs from the Database of Genomic Variants (Zarrei *et al.*[9]). We have supplied data on those loci that are most impactful on DNA methylation signal in a European population within disease related regions.

We wish to convey that these genetically driven differences in signal show considerable and very defined population variability. Furthermore, they have the potential to substantially impact on allelic differences in regional and/or neighbouring

epigenetic state. Many of these effects will not be clearly apparent via DNA methylation array analysis, as our allelic analysis reveled. Most epigenetic modifications are assumed by many to be 'pure', therefore this survey dramatically increases the potential scale of obligatory, facilitated and dosage effects that exist across these robust disease associated regions for a more nuanced understanding of the human DNA methylome.

With regard to CpG-SNPs, the potential for these to facilitate GWAS-associated allelic epigenetic differences is a current research area of considerable interest. We have precisely delineated thousands of these regions of potential functional relevance as their genetic occurrence enables the potential of epigenetic variability. They may then vary in a tissue- or time-specific manor. Recently McClay *et al.* found CpG-SNPs to be specifically functionally enriched within GWAS regions only[10]. Additionally strong CpG-SNP effects were also seen in the Jaffe *et al.* paper referred to by the reviewer. Even single CpG-SNPs have been shown to have the potential to influence the entire promoter methylation state[7].

These population scale MeDIP-seq derived DNA methylomes give a significantly different picture of the epigenetic variability across the genome compared to scattered vision provided by arrays and these data may help the complex interpretation of array studies. Within these GWAS LD block regions the new 850k array for instance has 1,815 probes (~0.65% of the 278,873 probes within GWAS LD Block regions), which directly overlap an HSM peak and an additional 4,856 (1.74%) that are within 1kb. This is vital information for researcher using this methodology.

We have added the following to further clarify this point to the Abstract and Introduction:

Abstract:

"Allelic variation in non-SNP genetic variants, which contribute to non-epigenetic dosage effects, as well as CpG-SNPs are strongly implicated."

"These Haplotype-Specific DNA Methylation (HSM) peaks, whilst predominately not tissue-specific, do show functional enrichment, including for DNase-I Hypersensitivity sites, Enhancers and allele-specific CTCF."

"Our results demonstrate strong allelic differences in DNA methylation driven by obligatory or facilitative genetic effects, that may have direct or regional functional impact in these common disease-related regions. These require disentangling from pure tissue-specific modifications. Furthermore, these local effects may influence array-based studies, and imply underestimated population variability in current reference epigenomes."

Introduction:

"CpG density is fundamental in defining the background DNA methylome, along with cell-specific usage of available binding sequences for transcription factors[11]. Common

population variation in CpGs is a significant via SNPs within this dinucleotide (CpG-SNPs) as well as larger variants."

"This leads to a distinct signal of population variation in both facilitated and obligatory genetic effects on the DNA methylome. Direct genetic dosage effects on this DNA methylation score are allelic signal differences not epigenetic variability. However, these may as well contribute to regional or neighbouring allelic epigenetic variation, with effects on traits, or even bias other analytical measures."

It would be helpful to have more information on the HSM peaks. How long are they? Fig 4 illustrates such peaks but the font is so small that it is not possible to get any useful information from them. If the peaks are relatively short, e.g. within a single window of ~500 bp, do they still represent haplotypic effects? Or they are cases of meQTL or allele-specific methylation? In this context, some relevant papers which investigated meQTL in pan-GWAS context, e.g. Jaffe et al. Nature Neuroscience 2015 (or 2016), should be cited and discussed.

Yes we agree with the reviewer and have provided additional data regarding the HSM peaks, the average size is 0.82 kb as detailed in the Results section. We now included a graph of size as Supplementary Figure S2 (Figure R4 below). We have also now provided a website to browse the all the results graphs and download all the data – http://epigenome.soton.ac.uk/hsm/hsm.php

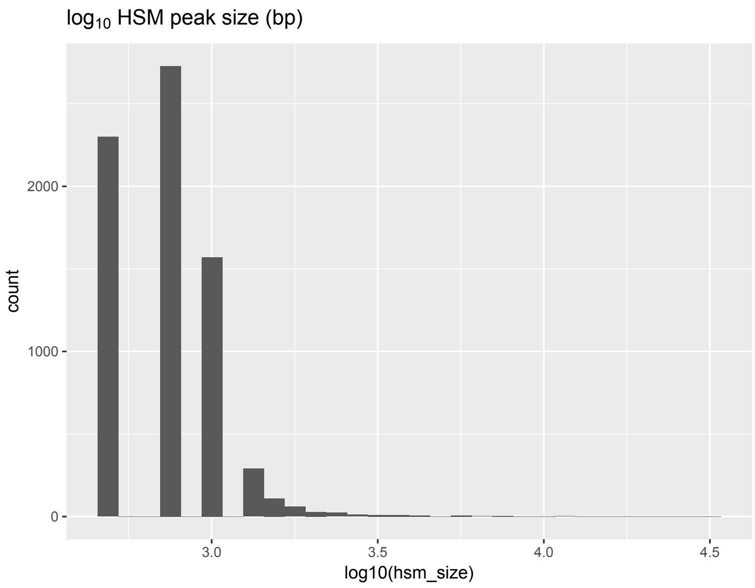

Figure R4: HSM peak size in log_{10} bp (now Supplementary Figure S2)

We have termed these results haplotypic as they are defined as differences between risk

and non-risk GWAS SNP haplotypes, defined by recombination rate boundaries. Whilst single SNPs could drive some of the HSM peaks, there evidence that there is an increased number of SNPs within these regions (6.60 SNPs/kb) compared to GWAS SNP LD Block regions (3.91 SNPs/kb). Additionally, there is a significant density increase in CpG-SNPs within HSM peaks as seen in Figure R5/Figure 5B discussed in the paper. 'Other' category HSM peaks are furthermore strongly enriched for multiple CpG-SNPs (see Figure R6 /Supplementary Figure S5). We have now included discussion of this and these results as below.

Results:

"There is also an increased number of SNPs within HSM peaks (6.60 SNPs/kb) compared to GWAS SNP LD Block regions (3.91 SNPs/kb, $p < 2.2 \times 10^{-16}$)."

"92.6% of 'Other' HSM peaks contain a CpG-SNP with an average of 2.26 CpG-SNPs within these peaks thus indicating that clusters of CpG-SNPs are strong drivers of HSM signal outside other known variants. (Supplementary Figure S5)."

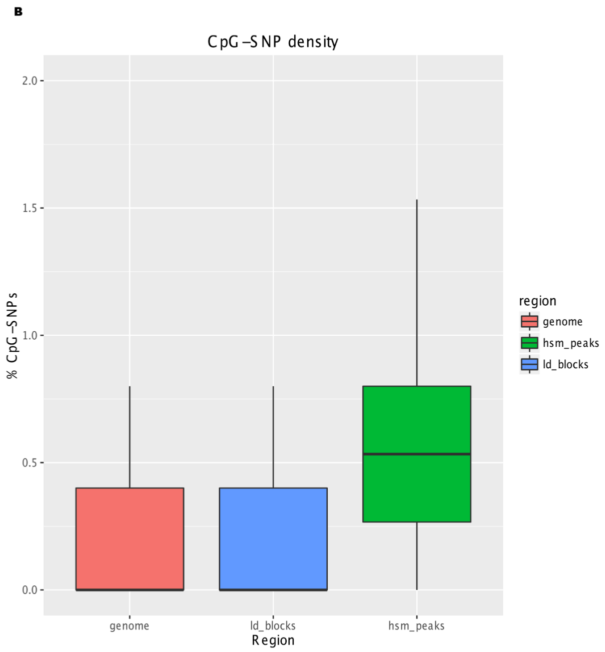

Figure R5 (and Figure 5B in paper):
CpG-SNP density (as %) compared across the genome, the GWAS LD Blocks and within the HSM Peaks.

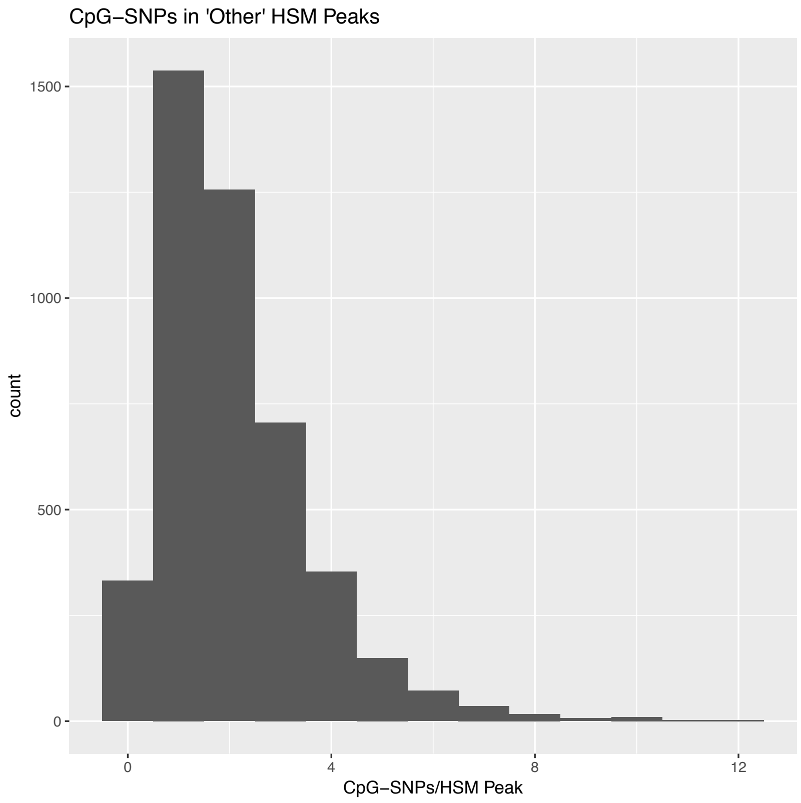

Figure R6 (and Supplementary Figure S5).
CpG-SNPs within 'Other' variant HSM peaks. CpG-SNPs clusters contribute to a majority, but not all, HSM peaks within the 'Other' category, *i.e.* do not overlap CNVs, Indels or STRs, or combinations of these.

We also thank the reviewer for pointing out the significant relevance of the Jaffe *et al.* paper to our work and we have now included this within the Discussion, as below.

Discussion:

"Previous work from Jaffe *et al.* [12] has also investigated GWAS loci with respect to DNA methylation from array data and identified a strong enrichment for mQTL within these loci. Furthermore, many of these mQTLs were not cell-type specific and involved critical CpG disrupting SNPs, in similar fashion to our HSM dataset. That analysis also proposed that many GWAS variants and highly-correlated proxy SNPs influence nearby DNA methylation and in this way impact upon disease risk"

1. Gibbs, J.R. *et al.* Abundant quantitative trait loci exist for DNA methylation and gene expression in human brain. *PLoS Genet* **6**, e1000952 (2010).
2. Gaunt, T.R. *et al.* Systematic identification of genetic influences on methylation across the human life course. *Genome Biol* **17**, 61 (2016).
3. Chen, L. *et al.* Genetic Drivers of Epigenetic and Transcriptional Variation in Human Immune Cells. *Cell* **167**, 1398-1414.e24 (2016).

4. Ziller, M.J. *et al.* Charting a dynamic DNA methylation landscape of the human genome. *Nature* **500**, 477-81 (2013).
5. Maurano, M.T. *et al.* Systematic localization of common disease-associated variation in regulatory DNA. *Science* **337**, 1190-5 (2012).
6. Lienert, F. *et al.* Identification of genetic elements that autonomously determine DNA methylation states. *Nature genetics* **43**, 1091-7 (2011).
7. Kumar, D. *et al.* A functional SNP associated with atopic dermatitis controls cell type-specific methylation of the VSTM1 gene locus. *Genome Medicine* **9**, 18 (2017).
8. Andrews, S.V., Ladd-Acosta, C., Feinberg, A.P., Hansen, K.D. & Fallin, M.D. "Gap hunting" to characterize clustered probe signals in Illumina methylation array data. *Epigenetics Chromatin* **9**, 56 (2016).
9. Zarrei, M., MacDonald, J.R., Merico, D. & Scherer, S.W. A copy number variation map of the human genome. *Nat Rev Genet* **16**, 172-183 (2015).
10. McClay, J.L. *et al.* High density methylation QTL analysis in human blood via next-generation sequencing of the methylated genomic DNA fraction. *Genome Biol* **16**, 291 (2015).
11. Schubeler, D. Function and information content of DNA methylation. *Nature* **517**, 321-326 (2015).
12. Jaffe, A.E. *et al.* Mapping DNA methylation across development, genotype and schizophrenia in the human frontal cortex. *Nat Neurosci* **19**, 40-7 (2016).

REVIEWERS' COMMENTS:

Reviewer #1 (Remarks to the Author):

The authors have been thoughtful and highly responsive to review. I think the paper is now much more readable and presents important data and insights. I also appreciate the detailed response to reviews and think the readership will benefit from the publication of this review process.

There remain a few typos, but these can be corrected in editorial review. I think this will be an important contribution and support its publication.

Reviewer #3 (Remarks to the Author):

In response to reviewers' comments, the authors say that the obligatory (and probably strong facilitative) genetic effects should be observed in all tissue, which seems like a reasonable assumption. They also show that there is no bias towards HSM enrichment in blood derived DNase-1 hypersensitivity sites in ENCODE. Therefore, the results from whole peripheral blood seems appropriate.

REVIEWERS' COMMENTS:

Reviewer #1 (Remarks to the Author):

The authors have been thoughtful and highly responsive to review. I think the paper is now much more readable and presents important data and insights. I also appreciate the detailed response to reviews and think the readership will benefit from the publication of this review process.

There remain a few typos, but these can be corrected in editorial review. I think this will be an important contribution and support its publication.

We thank the reviewer for their positive comments on our paper. We have agreed for the reviews to be fully available to the readership.

Reviewer #3 (Remarks to the Author):

In response to reviewers' comments, the authors say that the obligatory (and probably strong facilitative) genetic effects should be observed in all tissue, which seems like a reasonable assumption. They also show that there is no bias towards HSM enrichment in blood derived DNase-1 hypersensitivity sites in ENCODE. Therefore, the results from whole peripheral blood seems appropriate.

We thank the reviewer and consider attention to these obligatory and facilitative genetic effects is important in the interpretation of epigenomic data.
